# RNA Editing in Glioma as a Sexually Dimorphic Prognostic Factor That Affects mRNA Abundance in Fatty Acid Metabolism and Inflammation Pathways

**DOI:** 10.3390/cells11071231

**Published:** 2022-04-05

**Authors:** Sheng-Hau Lin, Sean Chun-Chang Chen

**Affiliations:** Graduate Institute of Biomedical Informatics, College of Medical Science and Technology, Taipei Medical University, Taipei 11031, Taiwan; seanlinabroad@gmail.com

**Keywords:** RNA editing, glioblastoma, survival, machine learning, sexual dimorphism, ADAR, PKR

## Abstract

RNA editing alters the nucleotide sequence and has been associated with cancer progression. However, little is known about its prognostic and regulatory roles in glioma, one of the most common types of primary brain tumors. We characterized and analyzed RNA editomes of glioblastoma and isocitrate dehydrogenase mutated (IDH-MUT) gliomas from The Cancer Genome Atlas and the Chinese Glioma Genome Atlas (CGGA). We showed that editing change during glioma progression was another layer of molecular alterations and that editing profiles predicted the prognosis of glioblastoma and IDH-MUT gliomas in a sex-dependent manner. Hyper-editing was associated with poor survival in females but better survival in males. Moreover, noncoding editing events impacted mRNA abundance of the host genes. Genes associated with inflammatory response (e.g., *EIF2AK2*, a key mediator of innate immunity) and fatty acid oxidation (e.g., *acyl-CoA oxidase 1*, the rate-limiting enzyme in fatty acid β-oxidation) were editing-regulated and associated with glioma progression. The above findings were further validated in CGGA samples. Establishment of the prognostic and regulatory roles of RNA editing in glioma holds promise for developing editing-based therapeutic strategies against glioma progression. Furthermore, sexual dimorphism at the epitranscriptional level highlights the importance of developing sex-specific treatments for glioma.

## 1. Introduction

Glioma, the most common primary brain tumor in adults, has an annual incidence rate of nearly six cases per 100,000 worldwide [1]. The aggressiveness of gliomas varies (WHO grade 2, 3, or 4) [2]. Patients with lower grade glioma (LGG; grade 2 and 3) typically have favorable outcomes than those with glioblastoma (GBM; grade 4) [3]. However, many LGG cases eventually progress to GBM [4]. With the standard of care (surgery, chemotherapy, and radiation therapy) [5], GBM remains incurable with a five-year survival rate of 5.1% [3]. The unresponsiveness to treatment arises from high levels of intratumor heterogeneity [6] and the poor understanding of molecular pathogenesis [7]. Genomic studies of GBM have revealed common genetic alterations in *EGFR* (*epidermal-growth factor receptor*), *MDM2* (*mouse double minute 2 homolog*), and *PTEN* (*phosphatase and tensin homolog*) [8,9]. Since these alterations would disturb cell cycle and signaling pathways, inhibitors targeting these pathways have been tested. However, these inhibitors exhibited limited efficacy [9].

Patient stratification is critical for better disease management and developing novel targeted therapies. Prognostic markers for GBM have been proposed, but they exhibited limited clinical utility. DNA methylation of *MGMT* (*O^6^-methylguanine-DNA methyltransferase*) promoter was a favorable prognostic factor for GBM in females only [10,11]. Moreover, conflicting results of expression-based subtyping of GBM (classical, proneural, and mesenchymal) [12] have been reported. Verhaak et al. [12] showed that proneural subtype exhibited prolonged survival, while Wang et al. [13] found that mesenchymal subtype had poor survival. Thus, identification of reliable prognostic markers that could be routinely used in clinical practice is of utmost importance.

Recent studies have revealed clinical relevance of adenosine to inosine (A-to-I) RNA editing in human cancers [14,15,16]. A-to-I RNA editing is a widespread post-transcriptional phenomenon that converts adenosine to inosine through Adenosine Deaminases Acting on RNA (ADARs) [17]. Nucleotide changes caused by RNA editing could alter protein sequence, RNA secondary structure, and microRNA-mediated regulation of mRNA abundance [18]. The link between RNA editing and carcinogenesis has been reported in several cancer types [15,16,19,20,21], indicating that the dynamic nature of RNA editing may help cancer cells to adapt to distinct disease states and/or microenvironments [22]. However, little is known about the roles of RNA editing in glioma progression. Galeano et al. observed reduced editing of *CDC14B* (*cell division cycle 14B*) in GBM [23], and Patil et al. reported that loss of editing of *GABRA3* (*gamma-amino butyric acid receptor alpha subunit 3*) facilitated glioma migration and invasion [24]. Nevertheless, the two studies compared editing differences between normal brain and glioma, providing little hint on the prognostic potential of RNA editing. Silvestris et al. analyzed editing profiles to stratify GBM patients [11]. However, this study included non-GBM samples, such as those with *isocitrate dehydrogenase* (*IDH*) mutation (now called WHO grade 4 astrocytoma [1]) and/or those belonging to the neural subtype (contamination of nontumor cells [13]). As such, whether RNA editing represents an independent prognostic marker for glioma awaits to be determined.

Furthermore, millions of editing events have been identified in human studies, but only a small number of them have been confirmed in functional studies. Particularly, these studies mostly focused on editing events on the coding regions [16,25,26], which represent less than 1% of the identified editing sites [15]. Although a few studies have investigated the regulatory potential of noncoding editing events on the transcriptome-wide scale [27,28,29], the regulatory role of RNA editing during glioma progression remains unclear.

Here, we introduce a novel pipeline that allows elucidating the prognostic and regulatory potential of RNA editing in glioma. Our risk stratification method reveals sexually dimorphic association between editing signatures and patient survival. Clinical utility of RNA editing is further validated by an independent GBM cohort. Supported by correlation analysis and *ADAR1* knockdown (KD) experiment, we show that survival-associated editing sites impact mRNA abundance of their host genes. Moreover, editing-regulated genes that are associated with glioma progression are enriched in inflammatory response and propanoate metabolism pathways. Among them, *EIF2AK2*, a key regulator of the innate immune response to viral infection, shows the maximum editing difference between high- and low-risk gliomas.

## 2. Materials and Methods

### 2.1. Clinical Information and Status of Known Glioma Biomarkers

For The Cancer Genome Atlas (TCGA) GBM and LGG samples, we downloaded information about sex, age, tumor origin (primary or secondary) and status (de novo or recursive), transcriptome subtype, mutation status of *IDH*, 1p/19q codeletion and *ATRX* (*alpha-thalassemia/mental retardation, X-linked*), methylation status of *MGMT* promoter and CpG island (G-CIMP-low or G-CIMP-high), and promoter mutation/expression of *telomerase reverse transcriptase* (*TERT*) from cBioPortal (https://www.cbioportal.org/; accessed 31 March 2019) [30]. Survival data (OS and PFI) were downloaded from the previous study [31]. For the Chinese Glioma Genome Atlas (CGGA) samples, information about sex, age, OS, tumor origin (primary or secondary), mutation status of *IDH*, and methylation status of *MGMT* promoter were downloaded from CGGA (http://www.cgga.org.cn/; accessed 20 February 2020) [32].

### 2.2. Prediction of Mutation Status of IDH and 1p/19q in TCGA Samples

For samples with unidentified mutation status of *IDH* and 1p/19q codeletion, we predicted their status by developing a methylation-based random forest (RF) model. DNA methylation data of Illumina 27 K array (Illumina Infinium Human Methylation 27 K BeadChip) were downloaded from Genomic Data Commons (GDC; https://portal.gdc.cancer.gov/; accessed 1 August 2019) [33]. The downloaded beta values were used to estimate methylation level, and the batch effect was controlled using quantile normalization [34]. The training dataset included 869 glioma samples with known mutation status of *IDH* and 1p/19q codeletion. As described in the previous study [35], we performed 10-fold cross validation (CV) along with feature selection, which achieved an area under the receiver operating characteristic curve of ~1.0 (Table S1).

### 2.3. Characterization of Editing Events of TCGA and CGGA Samples

RNA editing events of TCGA GBM and LGG samples were obtained from our previous study [36]. Briefly, our pipeline first followed Lo Giudice et al.’s protocol [37]. To reduce false positive calls derived from somatic mutations or germline SNPs, we only considered known editing sites stored in REDIportal, a well-known and the most up-to-date editing database [38]. Next, for each sample we excluded somatic mutations and non-recorded SNPs (i.e., germline mutations) identified by the TCGA network. We further controlled the issue of sequencing error by excluding sites whose editing level did not pass the binomial test (FDR > 0.05), assuming a sequencing error rate of 0.1% [36]. Discriminative editing sites were selected based on read coverage (A + G reads ≥ 10), evidence of editing (edited G reads ≥ 3 and editing level > 0.1% by binomial test with Benjamini–Hochberg-adjusted *p* value < 0.05) and variation in editing levels among patients (median of the absolute deviations from the data’s third quantile > 0) [35]. We downloaded RNA-Seq data of 83 CGGA primary GBM samples from the NCBI Sequence Read Archive (SRP027383 and SRP091303) [39] to detect their editing events. For a fair comparison, CGGA samples were analyzed similar to TCGA samples. Low-quality reads (quality score < 20) were first discarded using the NGS QC Toolkit [40]. Next, we obtained bam files by aligning reads to the GRCh38 genome with STAR [41] according to the TCGA mRNA Analysis Pipeline [42]. These bam files were then used to detect editing events on sites reported in REDIportal [38], similar to our previous analysis [36].

### 2.4. Dimension Reduction, Clustering, and Visualization of Editing Profiles

We obtained editing profiles of 153 GBM and 511 LGG samples from TCGA cohort. Among them, 15 GBM samples were removed because they were treated, had *IDH* mutation, or belonged to the NE subtype. We also excluded 14 GBM samples that had more than 20% of sites exhibiting low coverage. Moreover, we removed seven LGG samples that had extreme long PFI (>10 years) to reduce potential effects of unknown factors. In total, we analyzed editing profiles of 124 GBM samples (45 females and 79 males) and 504 LGG samples (94 IDH-wildtype, 242 IDH-MUT, and 168 IDH-MUT with 1p/19q codeletion) using UMAP (Uniform Manifold Approximation and Projection). UMAP, a novel manifold learning technique for dimension reduction and data visualization, captures local relationships within clusters and global relationships between clusters [43].

Glioma samples were then clustered based on their locations on the UMAP graph using the HDBSCAN (Hierarchical Density-Based Spatial Clustering of Applications) algorithm. HDBSCAN takes noise into account and find clusters based on densities rather than distance [44]. Density-based algorithms (e.g., HDBSCAN) are robust to outliers and outperform traditional clustering algorithms for clustering uncertain data [45]. We tuned hyperparameters (min_cluster_size, cluster_selection_epsilon, and min_samples) to improve clustering results and selected the clustering that had the lowest *p* value (log-rank test) for the KM plot.

### 2.5. Survival Analysis

Although TCGA was not prospectively designed for survival analyses, the survival plots for most cancer types were similar to other cohorts aiming for survival analyses [31]. TCGA OS was selected as the main clinical outcome endpoint for GBM. Instead of OS, PFI was selected as the main clinical outcome endpoint for LGG according to the suggestion of Liu et al., who systematically analyzed TCGA clinical data [31]. The association between editing-based subtyping and survival was evaluated by Kaplan–Meier (KM) curve along with log-rank test. Multivariate Cox’s proportional hazard model was used to control for covariates, including age, *MGMT* promoter methylation, *ATRX* mutation, and *TERT* expression/mutation.

### 2.6. Comparisons of Editing and Gene Expression between High-Risk and Low-Risk Cases

To discover differentially edited sites (DESs) associated with survival, we compared editing difference between high-risk and low-risk cases (based on editing-based subtyping) using the latter as the reference (G_1_ for female GBM, G_2_ for male GBM, ODI_1_ for female GH, I_1_ for female OD, and I_2_ for male GH). Accordingly, we had three comparisons for females (F1: G_2_ vs. G_1_; F2: GH vs. ODI_1_; F3: I_2_ vs. I_1_ (OD only)) and two comparisons for males (M1: G_1_ vs. G_2_ and M2: I_1_ vs. I_2_ (GH only)). For each comparison, we first excluded sites with small variation in editing levels among patients. Sites with the same editing levels in > 70% samples and editing variances at the bottom 20% were removed. Next, we removed sites with the absolute median difference ≤ 3%. Lastly, the Mann–Whitney U test was used to evaluate editing difference of the remaining sites. Multiple comparisons were corrected using the Benjamini–Hochberg (BH) procedure. Sites with adjusted *p* value < 0.05 were considered DESs, and genes with at least one DES were considered differentially edited.

We used the R *DESeq2* package [46] to detect differentially expressed genes (DEGs) for each comparison. Level-3 RNA-Seq raw read count data of TCGA samples were downloaded from GDC (https://portal.gdc.cancer.gov/; accessed 1 March 2018) [33]. Genes with adjusted *p* value < 0.05 were considered DEGs.

### 2.7. Evaluation of the Gene Overlap

To measure the similarity in patterns of editing changes across the five comparisons (F1–F3 and M1–M2), genes were ranked based on the degree of differential editing using the R *Rank-rank Hypergeometric Overlap (RRHO)* package [47]. For each comparison, genes with differential editing were ranked according to the significance (−log_10_(Mann–Whitney U *p*-value)) and direction of editing difference (median editing levels of high-risk cases − median editing levels of low-risk cases). Thus, genes at the top of the ranked list were hyper-edited in high-risk cases, whereas those at the bottom were hypo-edited in high-risk cases. For genes with multiple editing sites, the site with the most significant editing change was selected.

We also measured the overlap between genes with differential editing and those with differential expression for each comparison. DEGs were ranked according to the significance (−log_10_(DESeq2 *p*-value)) and direction of expression differences between high-risk and low-risk cases. Accordingly, genes at the top of the ranked list were more highly expressed in high-risk cases, while those at the bottom were more lowly expressed in high-risk cases.

### 2.8. Development and Validation of Editing-Based Classification Models

Using TCGA GBMs as the training set, we developed sex-specific RF models to classify CGGA GBMs that carried wildtype *IDH* (24 females and 48 males). Our approach included two steps: (1) feature selection and parameter tuning; (2) model development. In the first step, sites that exhibited significant editing difference between G_1_ and G_2_ (adjusted *p* value < 5 × 10^−4^ for females and <10^−5^ for males) and were shared with the CGGA cohort were selected as the initial features (852 for females and 403 for males). Next, for sites with high collinearity, only one was selected (resulting in 346 sites for females and 156 sites for males). Lastly, we performed feature selection and parameter tuning (number of estimators, maximum depth, minimum sample to split, maximum samples, maximum features, the number of features) by five-fold CV on the training set. The parameter sets with the smallest *p* value (log-rank test) were chosen. The importance of individual site was ranked according to the average feature importance from the five folds. In the second step, we developed RF classifiers using TCGA samples and sites that were selected in the previous step. Python’s scikit-learn library was used to build RF classifiers. The classifiers were used to predict the subtype (G_1_/G_2_) of CGGA samples. We evaluated the performance of our models by KM curve (log-rank test) and multivariate Cox regression (controlling for age and *MGMT* promoter methylation) analyses.

### 2.9. Over-Representation Analyses on Genes with DESs and DEGs

Over-representation analyses of Gene Ontology (GO) terms were conducted using the R *clusterProfile* package [48]. We also performed the QIAGEN Ingenuity Pathway Analysis (IPA) canonical pathway analysis. Multiple comparisons were corrected using the BH procedure.

### 2.10. Regression Analysis

RSEM (RNA-Seq by Expectation-Maximization) value for mRNA abundance of TCGA and CGGA samples were downloaded from CGGA (http://www.cgga.org.cn/; accessed 10 January 2021) [32]. For each DES, we assessed the correlation between editing level and mRNA abundance of the host gene by fitting a linear model of log-transformed mRNA abundance (RSEM value) against editing level. Age was included in the model as a confounding factor.

### 2.11. Analysis of U87 ADAR1 KD RNA-Seq Data

RNA-Seq raw read count data of U87 GBM cell lines were downloaded from the NCBI Gene Expression Omnibus under accession no. GSE28040 [49]. Three replicates of samples transfected with a siRNA that targets the *ADAR1* gene and three replicates of samples transfected with a control siRNA were compared using *DESeq2*. Genes with adjusted *p* value < 0.05 were considered differentially expressed.

## 3. Results

### 3.1. Sexually Dimorphic Association between Editing Profiles and Patient Survival

**Figure 1:**
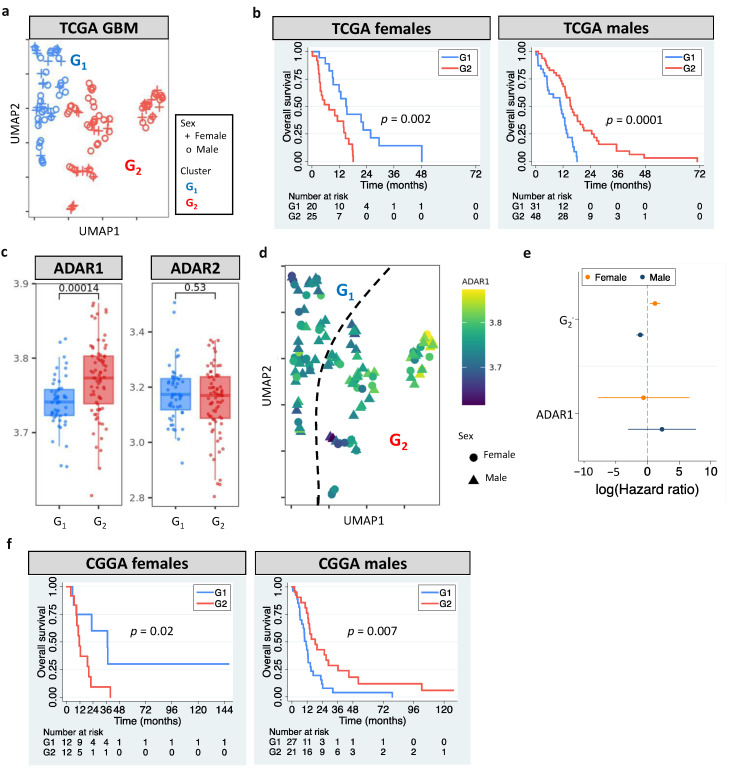
RNA editing-based subtyping of GBM samples. (**a**) UMAP of TCGA GBM samples (G_1_ and G_2_). (**b**) Kaplan–Meier (KM) curves for UMAP clusters (G_1_ and G_2_, log-rank test). (**c**) *ADAR1*/*ADAR2* expression of G_1_ and G_2_. (**d**) UMAP of GBM samples colored based on *ADAR1* expression. (**e**) Cox’s hazard ratios (HRs) of G_2_ and *ADAR1* expression for GBM survival. (**f**) KM curves for G_1_ and G_2_ of CGGA GBM samples. Dashed line indicates HR of 1.

The European Association of Neuro-Oncology updated the classification guidelines for adult glioma in 2020 [1]. GBM is now referred to WHO grade 4 gliomas that carry wildtype *IDH*. According to the update, we analyzed grade 4 gliomas with wildtype *IDH* in The Cancer Genome Atlas (TCGA). Our pipeline identified two clusters (G_1_ and G_2_, Figure 1a) by analyzing GBM editomes. The prognostic value of editing-based subtyping was evaluated in males and females separately since multiple lines of evidence have shown sex differences in GBM incidence and survival [50,51]. Intriguingly, samples in the same cluster exhibited sex dimorphism in survival. Compared to G_1_, G_2_ had poor overall survival (OS) in females but better OS in males (*p* = 0.002 for females and *p* = 0.0001 for males, Figure 1b). Sex difference in progression free interval (PFI) was also observed (*p* = 0.005 for females and *p* = 0.009 for males, Figure S1a). The trend persisted after controlling for confounding factors including age, *MGMT* promoter methylation, transcriptome subtype, and *TERT* expression/mutation. *ATRX* mutation status was not controlled because only one case had mutated *ATRX*. The hazard ratios (HRs) and 95% confidence interval (CI) of G_2_ for females and males, respectively, were 4.66 (1.12–18.13) and 0.36 (0.18–0.75). Interestingly, age and *MGMT* promoter methylation were prognostic in females but not in males (Table S2), highlighting the importance to include sex for the evaluation of prognostic markers.

The roles of RNA editors *ADAR1*/*ADAR2* in neuronal systems and brain disorders have been reported [52]. This prompted us to examine whether *ADAR1*/*ADAR2* expression correlated with editing-based subtyping. G_2_ had higher *ADAR1* expression than G_1_ (*p* = 0.00014), but no difference in *ADAR2* expression was observed (*p* = 0.53) (Figure 1c). Still, an unneglectable proportion of G_1_ and G_2_ exhibited similar *ADAR1* expression (Figure 1c,d), which likely resulted from other regulatory mechanisms of RNA editing [53]. For example, RNA-binding proteins have been shown to regulate RNA editing by interacting with *ADAR1* and binding to Alu elements of target mRNAs [54]. Multivariate cox regression further supported the prognostic value of RNA editing, not *ADAR1* expression (Figure 1e). These observations suggest that editing-based subtyping is independent of *ADAR1*/*ADAR2* expression.

**Figure 2:**
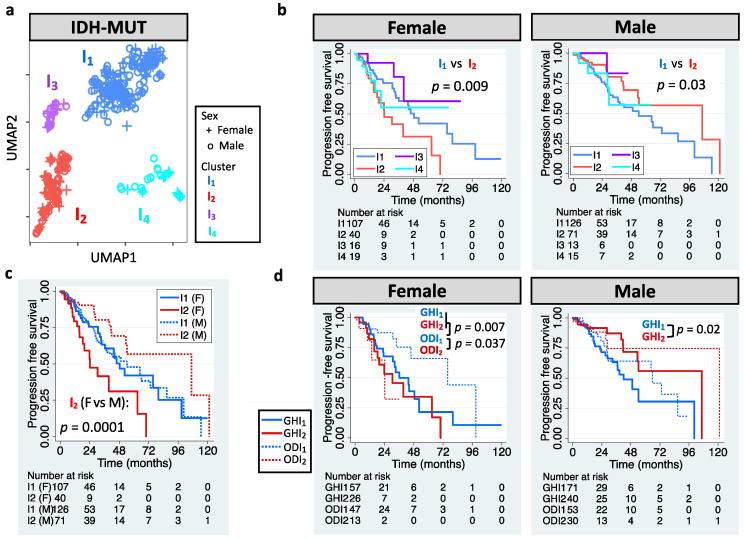
RNA editing-based subtyping of IDH-MUT samples. (**a**) UMAP of IDH-MUT gliomas (I_1_–I_4_). (**b**) Kaplan–Meier (KM) curves for UMAP clusters (I_1_–I_4_, log-rank test). (**c**) KM curves for I_1_ and I_2_. (**d**) KM curves for I_1_ and I_2_ of astrocytoma with high levels of DNA methylation (GH) and oligodendroglioma (OD).

The definition of LGG has also been updated. LGG is now referred to WHO grade 2 or 3 glioma that carry IDH mutation (herein called IDH-MUT glioma) [1]. We aimed to stratify IDH-MUT gliomas in TCGA LGG cohort. UMAP-based unsupervised clustering of editing profiles identified four clusters (I_1_–I_4_) for the 410 IDH-MUT gliomas (Figure 2a). Similar to GBM, IDH-MUT gliomas showed sex difference in survival with I_2_ acting like G_2_ (*p* = 0.009 for females and *p* = 0.03 for males; Figure 2b). The trend persisted after accounting for confounding factors, including age, *MGMT* promoter methylation, *ATRX* mutation, and *TERT* expression/mutation (HRs of I_2_: 2.07 [1.13–3.81] for females and 0.46 [0.25–0.86] for males). Note that editing-based subtyping was independent of expression signatures (Figure S1b). Intriguingly, sexually dimorphic survival arose from I_2_. Male and female I_1_ had similar survival (*p* = 0.95), but female I_2_ had poor survival compared to male I_2_ (*p* = 0.0001, Figure 2c).

Chromosome 1p/19q deletion (1p/19q codeletion) is the current marker for stratification of IDH-MUT gliomas [1]. Those without 1p/19q codeletion (astrocytoma, AS) have been found to have poor survival than those with 1p/19q codeletion (oligodendroglioma, OD) [1]. However, we found that only female AS showed poor PFI (*p* = 0.002 for females and *p* = 0.35 for males; Figure S1c). Thus, our approach outperformed the AS/OD classification for male IDH-MUT gliomas. To elucidate whether 1p/19q codeletion dictates the prognostic value of RNA editing, we analyzed AS and OD separately. Note that based on the levels of DNA methylation, two subtypes of AS have been identified, G-CIMP-high (GH) and G-CIMP-low (GL) [12,55]. Here, we focused on the GH subtype because it accounted for 95% of AS cases in TCGA. Moreover, it is crucial to identify cases with poor survival in GH because GH is considered to have better clinical outcome compared to GL [56]. We observed that I_1_/I_2_ successfully stratified male GH (*p* = 0.02) and female OD (*p* = 0.037), but not male OD (*p* = 0.26) or female GH (*p* = 0.34) (Figure 2d). The results remained when controlling for age and *ATRX* mutation (HRs of I_2_: 0.41 [0.19–0.89] for male GH and 3.82 [1.07–13.62] for female OD). Moreover, female GH and female ODI_2_ had similar survival (*p* = 0.35), and both had worse outcomes compared to female ODI_1_ (*p* = 0.007 for GH and 0.037 for ODI_2_, Figure 2d). The above results reveal that editing profiles stratify IDH-MUT gliomas in a sex- and 1p/19q codeletion-dependent manner.

To sum up, RNA editing profiles identified clinically relevant subgroups of gliomas. Our findings indicate clinical potential of integrating editing profiles and sex into the classification guidelines for both GBM and IDH-MUT tumors.

### 3.2. Editing Changes Are Shared between Sexes and Subtypes and Distinct from Differential Expression

**Figure 3:**
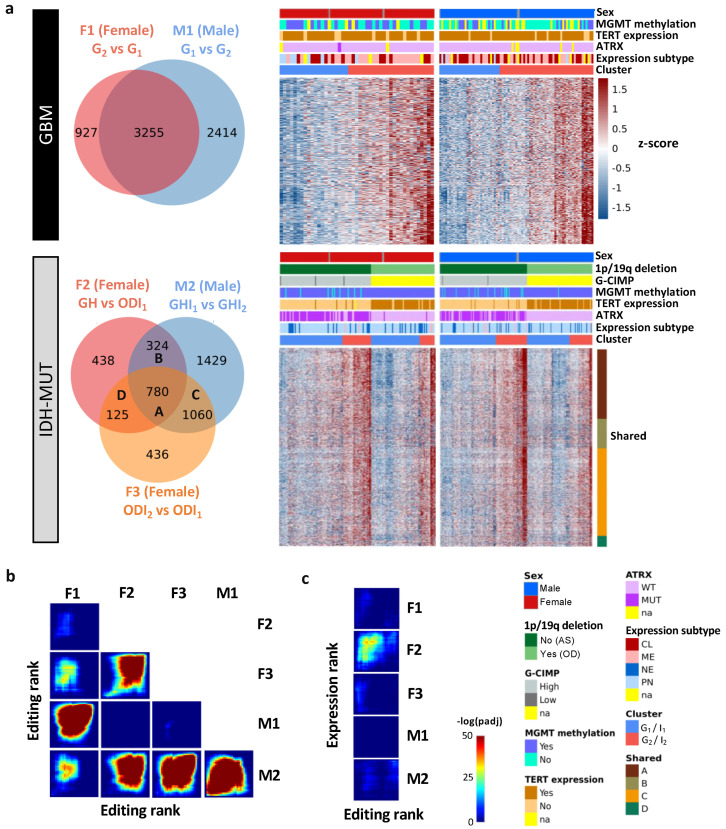
Editing changes during glioma progression. (**a**) Venn diagram and editing levels of differentially edited sites (DESs) between high- and low-risk gliomas. Comparisons are made for GBM (F1: G_2_ vs. G_1_ and M1: G_1_ vs. G_2_) and IDH-MUT gliomas (F2: GH vs. ODI_1_; F3: ODI_2_ vs. ODI_1_ and M2: GHI_1_ vs. GHI_2_). GH: astrocytoma with high levels of DNA methylation; OD: oligodendroglioma. (**b**) Overlap in genes with DESs between sexes and between comparisons. (**c**) Overlap between genes with DESs and those with differential expression in the five comparisons.

To explore editing difference between high-risk and low-risk cases, we identified differently edited sites (DESs) using low-risk cases (i.e., G_1_ and ODI_1_ for females; G_2_ and I_2_ for males) as the reference. The analysis generated three female DES sets (F1: G_2_ vs. G_1_; F2: GH vs. ODI_1_; F3: ODI_2_ vs. ODI_1_) and two male DES sets (M1: G_1_ vs. G_2_ and M2: GHI_1_ vs. GHI_2_) (Figure 3a). Based on these DES sets, G_2_ and I_2_ on average had higher editing levels (Figure 3a). Thus, high-risk gliomas were hyper-edited in females but hypo-edited in males, suggesting that RNA editing may exert sexually dimorphic effects on glioma progression.

We next asked whether differentially edited genes were shared between sexes and subtypes. We used the RRHO package [47] to measure overlaps in differentially edited genes across comparisons (F1–F3 and M1–M2). Genes were ranked according to the significance and direction of editing differences in each comparison. We observed significant gene overlaps between GBM and IDH-MUT (F1/F3, F2/F3, and M1/M2) and between sexes (F1/M1, F1/M2, F2/M2, and F3/M2) (Figure 3b). The overlaps indicate that editing alterations during glioma progression may disturb common pathways of GBM and IDH-MUT tumors and of both sexes.

To test whether differential gene expression accounted for differential editing, we also examined the overlap between differentially expressed genes (DEGs) and genes with differential editing. We used DESeq2 [46] to identify DEGs in F1–F3 and M1–M2 (Figure S2). No significant overlap was found, except for F2 (Figure 3c). Since F2 identified a large number of DEGs (n = 14,471), GH and OD may express entirely distinct sets of genes. Different expression programs between GH and OD may confine the detection of differential editing and differential expression simultaneously. Thus, editing changes are mostly independent of expression differences, indicating that differential editing is another layer of molecular alterations during glioma progression.

### 3.3. Clinical Utility of RNA Editing for GBM Prognosis Is Independently Validated

To validate our findings, we developed sex-dependent, editing-based random forest models to classify Chinese Glioma Genome Atlas (CGGA) GBM samples. Consistent with the observation in TCGA, CGGA G_2_ had poor OS in females but better OS in males (relative to G_1_, *p* = 0.02 for females and *p* = 0.007 for males, Figure 1f). The trend remained when controlling for age and *MGMT* methylation (HRs of G_2_: 3.9 [1.2–12.9] for females and 0.44 [0.23–0.85] for males). Our results demonstrate that RNA editing is an independent prognostic factor for GBM.

### 3.4. Differentially Edited Genes Are Associated with Immune Regulation and Cancer Progression

**Figure 4:**
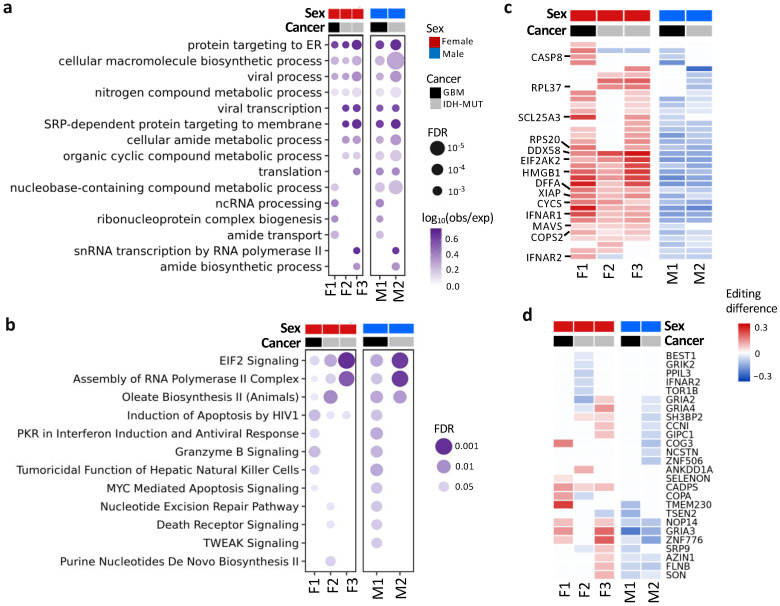
Functions and pathways associated with genes exhibiting differential editing. (**a**) Gene ontology enrichment analysis. (**b**) QIAGEN Ingenuity Pathway Analysis canonical pathway analysis. (**c**) Editing differences of selected genes associated with functions in (**a**) or (**b**). (**d**) Editing differences of sites in the coding regions. Comparisons are made between high- and low-risk GBM (F1: G_2_ vs. G_1_ and M1: G_1_ vs. G_2_) and IDH-MUT gliomas (F2: GH vs. ODI_1_; F3: ODI_2_ vs. ODI_1_ and M2: GHI_1_ vs. GHI_2_).

To detect pathways affected by differential editing during glioma progression, we performed over-representation analysis (ORA) on genes with differential editing, in contrast with those without differential editing (Figure 4a,b). ORA revealed pathways associated with immune regulation, protein targeting to ER, amide metabolism and transport, EIF2 signaling, and others (Tables S3 and S4). For example, G_2_/I_2_ showed hyper-editing of type 1 interferon (IFN) receptors (*IFNAR1* and *IFNAR2*), type 1 IFN-stimulated genes (ISGs) (e.g., *EIF2AK2*, *DDX58*/RIG-1, *MAVS*, *TRIM56*, and *TRIM69*) and genes involved in immune responses to viral infection (*EIF2AK2*, *DDX58*/RIG-1, *MAVS*, *CASP8*, *CYCS*, and *HMGB1*) [57,58] (Figure 4c).

Additionally, 26 genes had coding DESs (Figure 4d), and most of them have been shown to participate in tumor growth and metastasis (Table S5). For example, *NOP14* promoted proliferation and metastasis of pancreatic cancer cells [59], and loss of *CADPS* was associated with poor prognosis of malignant embryonal brain tumors [60]. Moreover, editing events of these genes have been associated with cancer progression (e.g., *AZIN1* [16], *COPA* [61], *CCNI* [62], and *FLNB* [63]) or neurological disorders (e.g., *GRIK2* and *GRIA2–4*) [52].

Together, genes involved in IFN response, inflammation, cancer cell proliferation and metastasis, and neuronal function were differentially edited during glioma progression.

### 3.5. Noncoding Editing Events Impact mRNA Abundance of Their Host Genes

**Figure 5:**
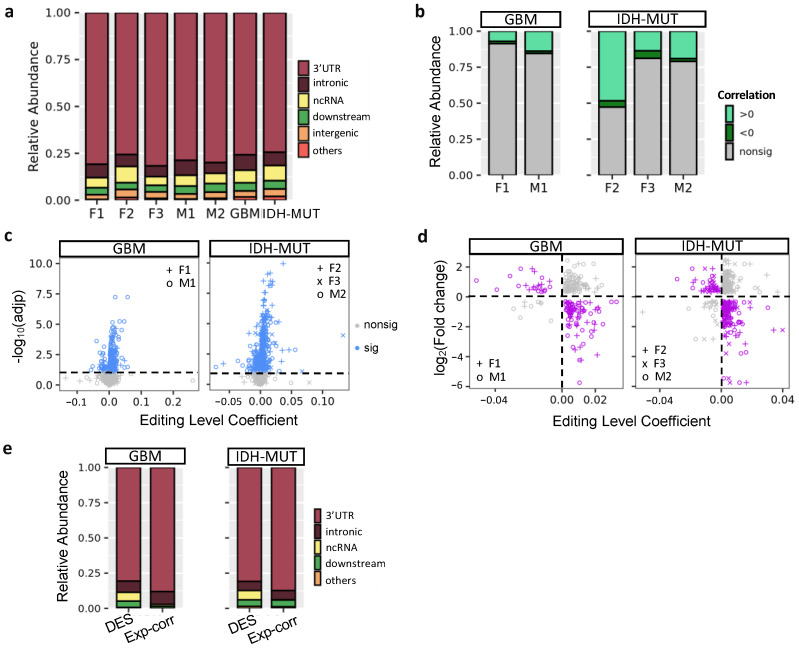
Regulatory potential of RNA editing in mRNA abundance. (**a**) Location of differentially edited sites (DESs) between high- and low-risk gliomas. Comparisons are made for GBM (F1: G_2_ vs. G_1_ and M1: G_1_ vs. G_2_) and IDH-MUT gliomas (F2: GH vs. ODI_1_; F3: ODI_2_ vs. ODI_1_ and M2: GHI_1_ vs. GHI_2_). (**b**) Distribution of correlations between mRNA abundance and RNA editing levels of DESs. (**c**) Scatter plot of coefficient and significance of editing level as a predictor of mRNA abundance, controlling for age. For genes with multiple sites associated with mRNA abundance, the one with the smallest adjusted *p* value was selected. Dashed line represents significance threshold at 10% false discovery rate. (**d**) Scatterplot of editing level coefficient estimate from (**c**) and fold change of the corresponding gene upon *ADAR1* KD cells. Purple points represent the direction of expression alterations in line with the sign of the editing association, whereas gray points represent the opposite. (**e**) Editing sites correlated with expression (Exp-corr) tend to locate in the 3′ UTRs, compared to all DESs (excluding intergenic ones).

Previous research showed that A-to-I editing mainly occurred in intronic regions [64,65]. However, ANNOVAR (20210501 version; gencode v.24) annotation [66] revealed that the majority of glioma DESs were located in the 3′UTRs (76–82%), followed by intronic regions (6–8%) and ncRNAs (5–9%) (Figure 5a). Because ~80% of glioma DESs were in 3′UTRs, we tested whether editing of these sites influenced mRNA abundance of their host genes. First, we calculated the correlation between editing levels of DESs and mRNA abundance of their host genes using linear regression controlling for age. A low fraction (9–21%) of DESs showed significant correlations, expect F2 (53%) (FDR < 10%, Figure 5b). On the gene level, we observed 273 (GBM) and 352 (IDH-MUT) genes, whose editing levels were correlated with mRNA abundance (Figure 5c).

Next, we investigated the impact of RNA editing on mRNA abundance by examining the transcriptome change of human U87 GBM cells upon *ADAR1* KD [49]. If a gene is regulated by RNA editing, *ADAR1* KD would alter its mRNA abundance. It would also exhibit inverse relationship between expression change and the coefficient of expression-editing correlation. Based on the two criteria, we identified 82 and 121 putative editing-regulated genes in GBM and IDH-MUT, respectively (Figure 5d and Table S6). We observed that expression-correlated DESs were enriched in 3′UTRs compared to all DESs of editing-regulated genes (*p* = 1.8 × 10^−4^ for GBM and *p* = 1.3 × 10^−4^ for IDH-MUT, Figure 5e), supporting the regulatory potential of expression-correlated DESs. Additionally, the majority of expression-correlated DESs had positive expression-editing correlations (72–92%, Figure 5b). Their host genes were also prone to show reduced expression upon *ADAR1* KD (77% were reduced, *p* < 10^−^^6^ for both GBM and IDH-MUT, binomial test). These observations suggest that RNA editing may regulate mRNA abundance through stabilization of mRNAs.

### 3.6. Necroptosis and Propanoate Metabolism Genes Are Editing-Regulated and Associated with Glioma Progression

**Figure 6:**
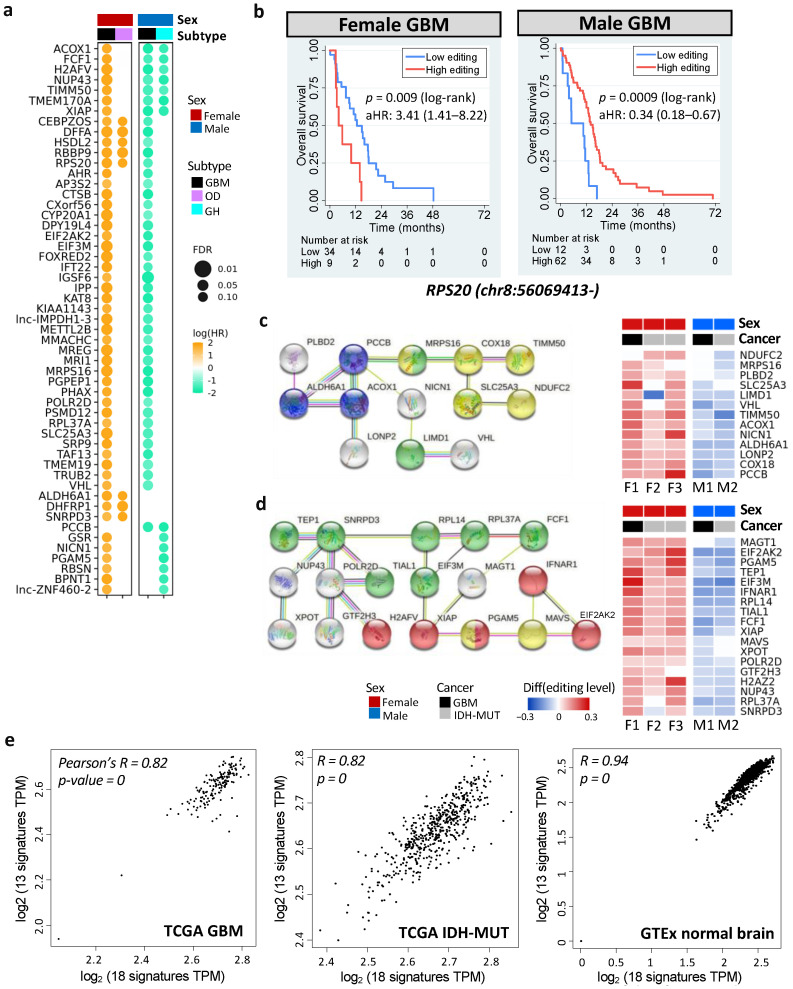
Prognostic and editing-regulated genes in glioma. (**a**) Age-adjusted hazard ratios (aHRs) of prognostic genes shared by both sexes or at least two cancer types (FDR < 0.15). GH: astrocytoma with high levels of DNA methylation; OD: oligodendroglioma. (**b**) Kaplan–Meier curves and aHR for editing levels of chr8:56069413- (*RPS20*), which is prognostic in both female and male GBM tumors. High vs. low: top 30% vs. bottom 70% for females and top 70% vs. bottom 30% for males. (**c**,**d**) represent PPI networks (left) and editing differences between high- and low-risk gliomas (right) of prognostic, editing-regulated genes. Comparisons are made for GBM (F1: G_2_ vs. G_1_ and M1: G_1_ vs. G_2_) and IDH-MUT gliomas (F2: GH vs. ODI_1_; F3: ODI_2_ vs. ODI, and M2: GHI_1_ vs. GHI_2_). Colors indicate enriched biological process (blue: propanoate metabolism, FDR = 0.0008; red: necroptosis, FDR = 0.0001) and cellular localization (yellow: ribonucleoprotein complex, FDR = 0.003; green: mitochondrial membrane, FDR = 0.006). (**e**) Scatterplots of gene expressions of the two networks in TCGA GBM tumors (left), TCGA IDH-MUT tumors (middle), and normal brain tissues from GTEx (right). X-axis represents genes in (**d**) and y-axis represents genes in (**c**).

To discover actionable biomarkers and potential therapeutic targets, we evaluated the prognostic value of DESs using Cox’s regression analysis accounting for age. We identified 378 prognostic DESs (and 197 genes) in GBM and 49 prognostic DESs (and 43 genes) in IDH-MUT, which showed sex disparities in prognosis (FDR < 0.15, Figure 6a and Figure S3). Among them, 22 genes were shared by GBM and IDH-MUT (Figure 6a).

Six prognostic DESs were shared by male and female GBM, including chr1:45509673+ (*MMACHC*), chr7:44832906- (*H2AFV*), chr8:56069413- (*RPS20*), chrX:119538709- (*STEEP1*), chr19:13773078+ (*MRI1*), and chr19:18366951+ (*PGPEP1*). Remarkably, these genes have been linked to cancer progression (Table S7). For example, elevated expression of RPS20 was associated with poor survival in GBM [67]. However, we observed that higher editing levels of *RPS20* showed worse prognosis in females (age-adjusted HR = 3.41 [1.41–8.22]), but better prognosis in males (age-adjusted HR = 0.34 [0.18–0.67]) (Figure 6b). The other five DESs displayed the same trend (Figure S4).

To discover functional modules of these prognostic genes, we constructed their protein–protein interaction (PPI) network using the STRING database [68]. Among them, 117 genes were connected, suggesting that these prognostic genes were biologically connected (PPI enrichment *p* = 3.7 × 10^−15^, Figure S5). ORA, on the 117 connected genes, identified pathways associated with necroptosis (regulated necrosis), the regulation of hypoxia-inducible factor-alpha, metabolism of amide and nitrogen compound, gene regulation, translational initiation, and others (FDR < 0.05, Table S8 and Figure S5). Furthermore, they tend to be regulated by *TP53* (FDR = 0.04), and their RNA secondary structures were prone to be altered by RNA editing (e.g., *XIAP* and *MAVS*, FDR = 0.005, Table S8) [69]. Note that necrosis and hypoxia are two hallmarks of GBM [70]. Our results were consistent with previous findings that the necrotic patterns predicted GBM survival [71] and that hypoxia was involved in glioma migration and invasion [72].

Next, we examined genes that were both prognostic and editing-regulated (n = 68). Among them, two PPI networks were found (PPI enrichment *p* = 0.002). One was associated with propanoate metabolism (FDR = 0.0008, Figure 6c) and the other was associated with necroptosis (FDR = 0.0001, Figure 6d). Genes in the two networks showed high expression correlations in TCGA gliomas (Pearson’s r = 0.82, *p* < 10^−10^) and GTex normal brain tissues (Pearson’s r = 0.94, *p* < 10^−10^) (Figure 6e, calculated by GEPIA: http://gepia2.cancer-pku.cn/#correlation; accessed 7 September 2021) [73]. Moreover, two networks became connected when including one of the prognostic genes (*AJUBA*, *ATM*, *STK4*, *UBB*, *MRTO4*, *RPS20*, *RPL23*, *RPL27A*, and *RPL7L1*) or editing-regulated genes (*CYCS*, *MDM2*, *POLR1A*, *DHTKD1*, and *H6PD*). Our observations revealed coordinated epi-transcriptional and transcriptional regulations of necroptosis and propanoate metabolism, indicating cross-talk between these pathways during glioma progression.

Editing levels of genes in the two networks displayed sex disparity in survival (Figure 6c,d and Table S9). They were also prognostic (Table S10) and editing-regulated (Table S11) in CGGA samples. Note that both necroptosis and propanoate play a role in inflammation [74,75,76], which is critical for cancer progression [77]. Thus, RNA editing may modulate glioma progression via regulating mRNA abundance of necroptosis and propanoate metabolism pathways.

### 3.7. EIF2AK2 Shows the Maximum Editing Difference between High- and Low-Risk Gliomas

**Figure 7:**
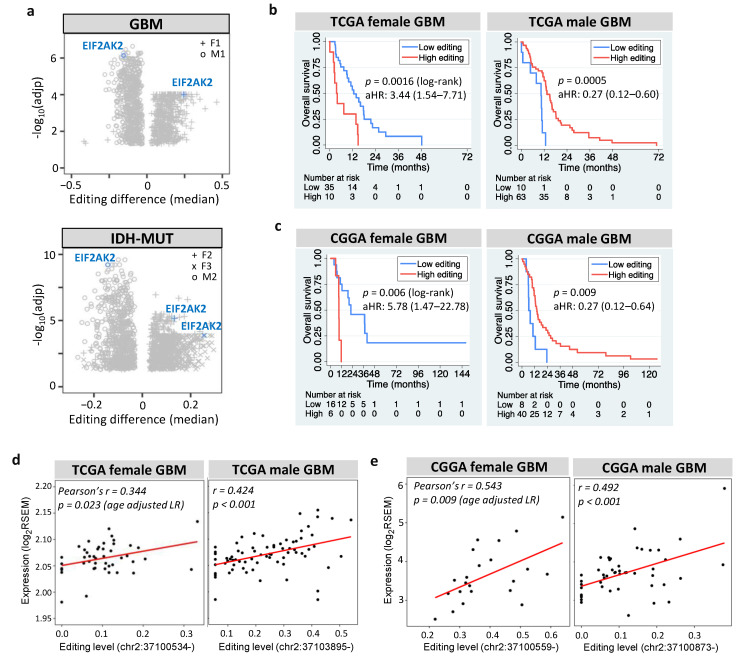
Prognostic value and editing-expression correlation of *EIF2AK2*. (**a**) Editing differences of *EIF2AK2* between high- and low-risk gliomas. Comparisons are made for GBM (F1: G_2_ vs. G_1_ and M1: G_1_ vs. G_2_) and IDH-MUT gliomas (F2: GH vs. ODI_1_; F3: ODI_2_ vs. ODI_1_ and M2: GHI_1_ vs. GHI_2_). (**b**,**c**) display Kaplan–Meier curves for editing levels of *EIF2AK2* in TCGA and CGGA GBM samples, respectively. High vs. low: top 30% vs. bottom 70% for females and top 70% vs. bottom 30% for males. aHR: age-adjusted hazard ratio. (**d**) and (**e**) represent scatterplots of editing level and mRNA expression of *EIF2AK2* in TCGA and CGGA GBM samples, respectively.

Among prognostic and editing-regulated genes, *EIF2AK2* showed the maximum editing difference between high- and low-risk gliomas (Figure 7a). *EIF2AK2* encodes PKR an IFN-induced protein kinase, which mediates the innate immune response to viral infection. *EIF2AK2* also plays a key role in angiogenesis and apoptosis [78], both of which are hallmarks of cancer [77]. We found that *EIF2AK2* exhibited sexually dimorphic prognosis of GBM in TCGA (Figure 7b) and CGGA (Figure 7c), consistent with both its anti- and pro-tumor effects [78]. The role of *EIF2AK2* in glioma is further supported by the finding that PKR activates nuclear factor-kappa B (NF-κB), which is essential for GBM growth [79]. Furthermore, *EIF2AK2* exhibited positive expression-editing correlations in GBM (Figure 7d for TCGA and Figure 7e for CGGA) and IDH-MUT gliomas (Figure S6). This agrees with the finding that editing of the 3′UTR of *EIF2AK2* increased endogenous *EIF2AK2* mRNA abundance in non-small cell lung carcinoma [27]. Collectively, RNA editing of *EIF2AK2* may function as a sex-dependent mediator of glioma progression through regulating the mRNA abundance.

## 4. Discussion

The poor understanding of molecular pathogenesis of glioma presents challenges for rational trial designs. For developing rational therapy recommendations, patient stratification is critical because the results of clinical trials could be incorrectly effective by including patients with favorable outcome. Considering that GBM remains incurable, it is crucial to discover prognostic factors. Stratification is also important for IDH-MUT gliomas. Although adjuvant therapy increases OS and PFI of IDH-MUT gliomas [80,81], it comes with adverse side effects [82]. It is, thus, beneficial to distinguish between high- and low-risk IDH-MUT gliomas; the former require immediate adjuvant therapy, whereas the latter may consider delayed therapy to diminish side effects [82].

We demonstrated the prognostic value of RNA editing for glioma in both sexes, unlike other prognostic biomarkers, which were mostly female-specific. DNA methylation of *MGMT* promoter was a favorable prognostic factor for female GBM only [11]. Wang et al. showed that the mesenchymal subtype exhibited poor survival [13], but we found that it was also female-specific (Figure S7a). Moreover, Silvestris et al. developed two editing-based approaches to stratify TCGA GBM patients, the Alu editing index (AEI) for males and editing profiles of 267 sites for females [11]. However, the prognostic value of AEI could not be confirmed after excluding IDH-MUT GBMs and/or using the most up-to-date clinical data from TCGA Pan-Cancer Clinical Data Resource [31] (*p* = 0.07, Figure S7b). The ambiguity likely resulted from the incomplete annotation of patient outcome in their study (e.g., missing subsequent follow-up data files) and the inclusion of IDH-MUT GBMs, which tend to have better prognosis. Furthermore, their editing-based approaches failed to stratify CGGA samples [11], greatly reducing the clinical utility of RNA editing. We further showed that the prognostic value of 1p/19q codeletion (the current biomarker for IDH-MUT gliomas) was limited to females (Figure S1c). In this study, we provided a robust, RNA editing-based approach for glioma stratification. We established its prognostic value by using the most complete prognostic information and controlling for expression changes and several confounding factors. The editing-based models that we developed were further validated in CGGA samples and can be routinely used in clinical practice.

Editing-based subtyping allows the identification of DESs during glioma progression and investigation of their functions. The observation that most DESs located in 3′UTRs prompted us to investigate the capacity of editing to modulate mRNA abundance. To date, three studies have investigated the regulatory potential of RNA editing on the transcriptome-wide scale [27,28,29]. Sharpnack et al. showed that 1413 genes displayed correlation between editing level and gene expression in lung adenocarcinoma [29], and Gu et al. discovered editing sites that could affect gene expression in four cancer types [28]. However, both studies did not analyze expression change upon *ADAR* KD. Chan et al. identified DESs between epithelial and mesenchymal tumors and found that DESs regulated mRNA abundance of their host genes by analyzing expression change upon *ADAR1* KD and conducting experimental validation [27]. Through analyzing glioma transcriptomes and expression change of the U87 GBM cell line upon *ADAR1* KD, we established the regulatory role of RNA editing in glioma. Some of the genes identified here have also been reported to be editing-regulated in lung adenocarcinoma [29] and/or during the epithelial–mesenchymal transition [27]. Moreover, some editing-regulated genes are mediators of tumor proliferation and migration and inflammatory response. Examples included *MGAT1* (mannosyl glycoprotein acetylglucosaminyl-transferase) for Wnt/β-catenin signaling, *GNA12* (G Protein Subunit Alpha 12) for RhoA/ROCK signaling and proinflammatory cytokine production, *IFNAR1* for JAK-STAT signaling, *EIF2AK2* and *MAVS* for innate immune response, and *XIAP* for inflammatory signaling. Note that although RNA editing affected mRNA abundance, genes with DESs did not significantly overlap with DEGs. Our observation agrees with the suggestion of Chan et al. [27] that differential editing may not necessarily cause significant expression differences because editing levels were relatively low.

Cumulated evidence has shown RNA editing as a key regulatory mechanism during tumorigenesis and cancer progression [83,84]. First, *ADAR1*-mediated A-to-I editing of *AZIN1* enhanced tumorigenesis in hepatocellular carcinoma [16] and esophageal squamous cell carcinoma [63]. Second, editing of *NEIL1* and *miR381* promoted the growth of A459 lung cancer cells [85]. Third, editing of *dihydrofolate reductase* (*DHFR*) increased its mRNA and protein abundance, which in turn enhanced cell proliferation and resistance to methotrexate in breast cancer [86]. Fourth, editing of the tumor suppressor *miR-200b* weakened its interaction with the target gene *ZEB1* in thyroid cancer, and the editing inhibitor 8-azaadenosine diminished aggressiveness of thyroid cancer cells [87]. Fifth, editing of *CDK13* increased its protein abundance and promoted cancer cell hallmarks in thyroid cancer [88]. Sixth, editing of *DOCK2* mRNA enhanced its stability and upregulated the expression of stemness and antiapoptotic genes, which in turn promoted oncogenesis of melanoma stem cells [89]. Lastly, editing of *let-7* pri-miRNA enhanced self-renewal of leukemia stem cells [90]. Altogether, RNA editing represents a novel oncogenic pathway in cancer development and progression.

We observed that editing profiles had sexually dimorphic prognostic values. Sex differences in incidence, disease phenotype, and clinical outcome have been well documented in GBM [50,91,92], but little is known about IDH-MUT gliomas. Moreover, the molecular differences that drive such different presentation and outcomes between sexes remain poorly understood. As shown in this paper and other studies [27,28,29], RNA editing acts as a novel regulatory mechanism for host gene expression. It is likely that gene expression has opposite effects in males and females instead of editing. Several lines of evidence have shown sexually dimorphic effect of gene expression on survival. For example, overexpression of glycolytic genes increased the survival in females but reduced the survival in males for GBM patients [93]. Expression-based clustering of GBM patients has also found a sexually dimorphic association between gene expression and patient survival [91]. Moreover, microglia and mast cells are brain-resident immune cells that modulate immune responses, and both are sexually dimorphic [94]. It has been shown that male and female microglia display divergent inflammatory responses to lipopolysaccharide [95]. Altogether, it is likely that RNA editing regulates mRNA abundance of genes that have a sexually dimorphic effect on glioma progression.

Metabolic reprogramming has emerged as an important mechanism to sustain tumor growth and survival [96]. Here, we showed that propanoate metabolism genes displayed sex disparity in the association between editing/expression and survival. Short-chain fatty acids, including propanoate (or propionate), acetate, and butyrate, control energy metabolism and supply through regulating glucose and lipid metabolism [97]. It has been suggested that males favor glucose and amino acid, while females prefer lipid for energy metabolism [98]. Furthermore, the roles of propionate in tumor development have been described, in which it suppressed proliferation, migration, and invasion of colon and lung cancer cells [99]. We showed that *ACOX1* (*acyl-CoA oxidase 1*), the rate-limiting enzyme in fatty acid β-oxidation, was prognostic and editing-regulated in glioma. Fatty acid oxidation has been shown to provide glioblastoma cells metabolic plasticity [100]. Additionally, inhibition of fatty acid oxidation improved GBM cell survival [101]. Together, RNA editing may modulate glioma progression on the basis of sex through the regulation of fatty acid oxidation (and propionate metabolism), which represents a key pathway for metabolic reprogramming and drives GBM tumorigenesis [102].

We also showed that IFN receptor (e.g., *IFNAR1*) and ISGs (e.g., *EIF2AK2* and *MAVS*) were prognostic and editing-regulated in glioma. The interaction of RIG-I and the *MAVS* protein results in the induction of the type I IFN and ISGs [58]. IFN drives necroptosis of macrophages [103], which ultimately increases the levels of pro-inflammatory cytokines (e.g., the IL-1 superfamily) [104]. Our results, thus, indicate the involvement of editing-regulated inflammatory response in glioma progression, consistent with the observation that IL1β blockade inhibited granulocytic monocytic myeloid-derived suppressor cells in female GBM patients [105]. Moreover, in addition to pro-inflammatory activity, necroptosis also has anti-inflammatory effects [106]. Since the immune system is involved in sex disparities in brain development [107], the above observations indicate that necroptosis may trigger sex-dependent inflammatory responses in the brain, consistent with our finding that editing of necroptosis genes exhibited sex-dependent prognosis.

Our findings agree with known physiological roles of RNA editing. *ADAR1*-mediated A-to-I RNA editing activates interferon and double-stranded RNA (dsRNA)-sensing pathways. Loss of editing was associated with mouse embryonic death, which was rescued by codeletion of *MDA5* or its downstream adaptor *MAVS* [108]. *MDA5* and *MAVS* mediate the process that marks unedited dsRNA as non-self [109]. These observations suggest that RNA editing of endogenous transcripts is essential for preventing cytosolic dsRNA response by self dsRNA [108] and suppresses innate immune stress responses [110]. Additionally, loss of RNA editing in *ADAR1*-deficient thymocytes reduced T cell receptor signal transduction, resulting in abnormal thymic T cell maturation [111]. Furthermore, dysregulated editing of serotonin 2C receptor led to growth retardation and reduced fat mass in mice [112]. In addition, editing of *GRIA2*, encoding GluA2 that is responsible for Ca2+ permeation and voltage rectification [113], plays an essential role in brain development and function [114]. Downregulation of *ADAR2* caused insufficient editing of *GRIA2*, leading to the death of motor neurons of sporadic amyotrophic lateral sclerosis patients [115]. Moreover, C-to-U RNA editing of *apolipoprotein B* (*APOB*), which modulates lipid metabolism, produces the APOB48 isoform in the small intestine by introducing a UAA stop codon [116]. Editing of *APOB* influenced plasma APOB levels and limited the deposits of intestinal lipoproteins in the arteries [117]. The above findings reveal diverse roles of RNA editing in immune response, brain development, and lipid metabolism.

To our knowledge, this study is the first to reveal the sex-dependent effect of *EIF2AK2* (PKR) on glioma progression. Previous studies have suggested the dual effects of PKR in inflammation and tumorigenesis [78]. In addition to the activation of pro-inflammatory pathways, PKR also triggers anti-inflammatory activity, such as IL-10 activation and reduced proliferation of CD8 T cells [78]. The anti-tumor role of PKR is supported by the observation that PKR overexpression inhibited cell growth [118] and that PKR stimulated apoptosis [119]. The tumor promotion role of PKR is established by reduced metastatic potential of murine melanoma upon PKR KD [120] and the inverse relationship between PKR expression and survival [78]. Moreover, PKR activates NF-κB, which is required for the glioblastoma growth [79] and has multiple roles in cancer development [121]. However, it has been suggested that NF-κB can be tumor promoting or anti-tumorigenic depending on tumor settings [122]. Collectively, PKR may act as a double-edged sword in glioma on the basis of sex, suggesting that PKR could be an attractive target for the treatment of glioma. Indeed, activation of PKR by a lentiviral vector inhibited GBM growth in mouse brain [123], and oncolytic virus that activated PKR signaling was evaluated in three Phase I trials in GBM patients [124].

We developed a stratification pipeline that incorporated RNA editing data in a novel manner by utilizing UMAP. We compared the results of UMAP to those of two other techniques, principal component analysis (PCA) and t-distributed stochastic neighbor embedding (t-SNE). Unlike UMAP, it was not straightforward to identify clusters from PCA and t-SNE plots (Figure S8). In PCA and t-SNE plots, cases of distinct UMAP clusters were overlapped, although those of the same UMAP cluster were locally aggregated (Figure S8). We also examined various sets of parameters for t-SNE, but we did not discover clusters with prognostic value. Our results agree with the observation that UMAP preserves more of the global structure [43], which is particular important for our purpose. Applying UMAP and HDBSCAN to our pipeline largely increases the performance. Moreover, our pipeline is applicable to other cancer types.

Note that it is uncertain whether editing differences identified here arose from cancer cells or other cell types (e.g., microglia or macrophages). Chan et al. [27] showed that cancer cells were the main cell type exhibiting editing difference between epithelial and mesenchymal lung tumors. Single-cell analysis of glioma editome will be required to clarify the contribution of distinct cell types to differential editing. Moreover, although we did not carry out experimental confirmation, nearly all of the necroptosis and propanoate metabolism genes and their interacting partners in Figure 6c,d (*MRPS16*, *VHL*, *LIMD1*, *NDUFC2*, *PCCB*, *ACOX1*, *LONP2*, *XIAP*, *RPL37A*, *EIF2AK2*, *PGAM5*, *MAVS*, *TIAL1*, *XPOT*, *NUP43*, *TEP1*, *SNRPD3*, *EIF3M*, and *MAGT1*) or their paralogs (*COX18/COX20*, *GTF2H3/GTF2H2*, *SLC25A3/SLC25A21*, *PLBD2/PLBD1*, *RPL14/RPL13*, *IFNAR1/IFNAR2*, and *POLR2D/POLR2A*) have been shown to be editing targets in HepG2 cells [69], lung cancer [29], or during epithelial-mesenchymal transition in several cancer types [27]. Thus, necroptosis and propanoate metabolism genes are likely true editing targets in glioma, although experimental confirmation is still needed.

## 5. Conclusions

In summary, we demonstrate RNA editing as a novel, sex-dependent prognostic factor in glioma, suggesting the importance of integrating editing profiles and sex into current classification guideline for better management of glioma. Our findings also highlight the extensive editing changes during glioma progression and their impact on mRNA abundance, especially for genes involved in inflammation and fatty acid oxidation. The above observations were confirmed in CGGA, indicating the clinical utility and therapeutic potential of RNA editing in treatment of glioma. In particular, *EIF2AK2* may acts as an editing-regulated mediator of glioma progression given its key role in inflammatory response.

## Data Availability

Raw RNA sequencing data are available at NCBI dbGaP (Accession Number phs000178), NCBI SRA (Accession Number SRP027383 and SRP091303), and NCBI GEO (Accession Number GSE28040). Other materials, including study protocol and statistical analysis, are available from the corresponding author on reasonable request.

## Supplementary Materials

The following supporting information can be downloaded at: https://www.mdpi.com/article/10.3390/cells11071231/s1, Figure S1: KM plots and UMAPs for editing-based subtyping of TCGA gliomas; Figure S2: Differentially expressed genes between high-risk and low-risk gliomas in TCGA; Figure S3: Age-adjusted hazard ratios of prognostic genes in TCGA gliomas; Figure S4: Kaplan–Meier curves for prognostic DESs shared by TCGA female and male GBM tumors; Figure S5: PPI network of 190 prognostic genes and selected pathways from ORA analysis of the 117 prognostic genes that were connected; Figure S6: Editing-expression correlations of *EIF2AK2* in TGGA IDH-MUT tumors; Figure S7: KM plots for TCGA GBM samples based on (a) transcriptome-based subtyping and (b) Alu editing index (AEI); Figure S8: Visualization of TCGA GBM samples using three dimension reduction techniques (UMAP, PCA, and t-SNE); Table S1: Confusion matrix and performance measures of random forest (RF) models that predict mutation status of IDH and 1p/19q in TCGA samples with 10-fold cross validation; Table S2: Hazard Ratios (HRs) and 95% confidence intervals (CIs) of covariates for overall survival of GBM patients using Cox regression; Table S3: IPA canonical pathway analysis on genes with differential editing (DE) and those without editing (nonDE); Table S4: Gene ontology analysis of genes with differential editing (DE) and those without editing (nonDE); Table S5: Cancer-associated functions of genes with coding DES in Figure 4d; Table S6: List of editing-regulated and/or prognostic genes in TCGA gliomas; Table S7: Functions of genes with prognostic DESs shared by male and female GBMs; Table S8: GO and KEGG analysis of 117 prognostic genes that were connected in the PPI network; Table S9: Editing sites and related information of genes editing-regulated, prognostic genes (Figure 6c,d) in TCGA; Table S10: HRs of editing-regulated, prognostic genes (Figure 6c,d) in CGGA; Table S11: Correlation of editing-regulated, prognostic genes (Figure 6c,d) in CGGA.
