# Supplementary material for: RNA Editing in Glioma as a Sexually Dimorphic Prognostic Factor That Affects mRNA Abundance in Fatty Acid Metabolism and Inflammation Pathways
Source: Cells. 2022 Apr 5;11(7):1231. doi: 10.3390/cells11071231 (PMC8997934; doi:10.3390/cells11071231)
Supplement: Supplementary file 1 [file cells-11-01231-s001.zip › cells-1611912-supplementary.pdf]

Supplementary material

RNA editing in glioma as a sexually dimorphic prognostic factor that affects mRNA abundance in fatty acid metabolism and inflammation pathways

Sheng-Hau Lin[1, 2] and Sean Chun-Chang Chen[1]

[1] Graduate Institute of Biomedical Informatics, College of Medical Science and Technology, Taipei Medical University, Taipei 11031, Taiwan.

[2] Department of Computer Science, Rice University, Houston, TX 77005, United States

Correspondence:
Sean Chun-Chang Chen
Graduate Institute of Biomedical Informatics, College of Medical Science and Technology, Taipei Medical University, No.250, Wu-hsing St., Taipei 11031, Taiwan;
Phone: +886-2-66382736#1512 Fax: +886-2-66380233
E-mail: seanchen@tmu.edu.tw

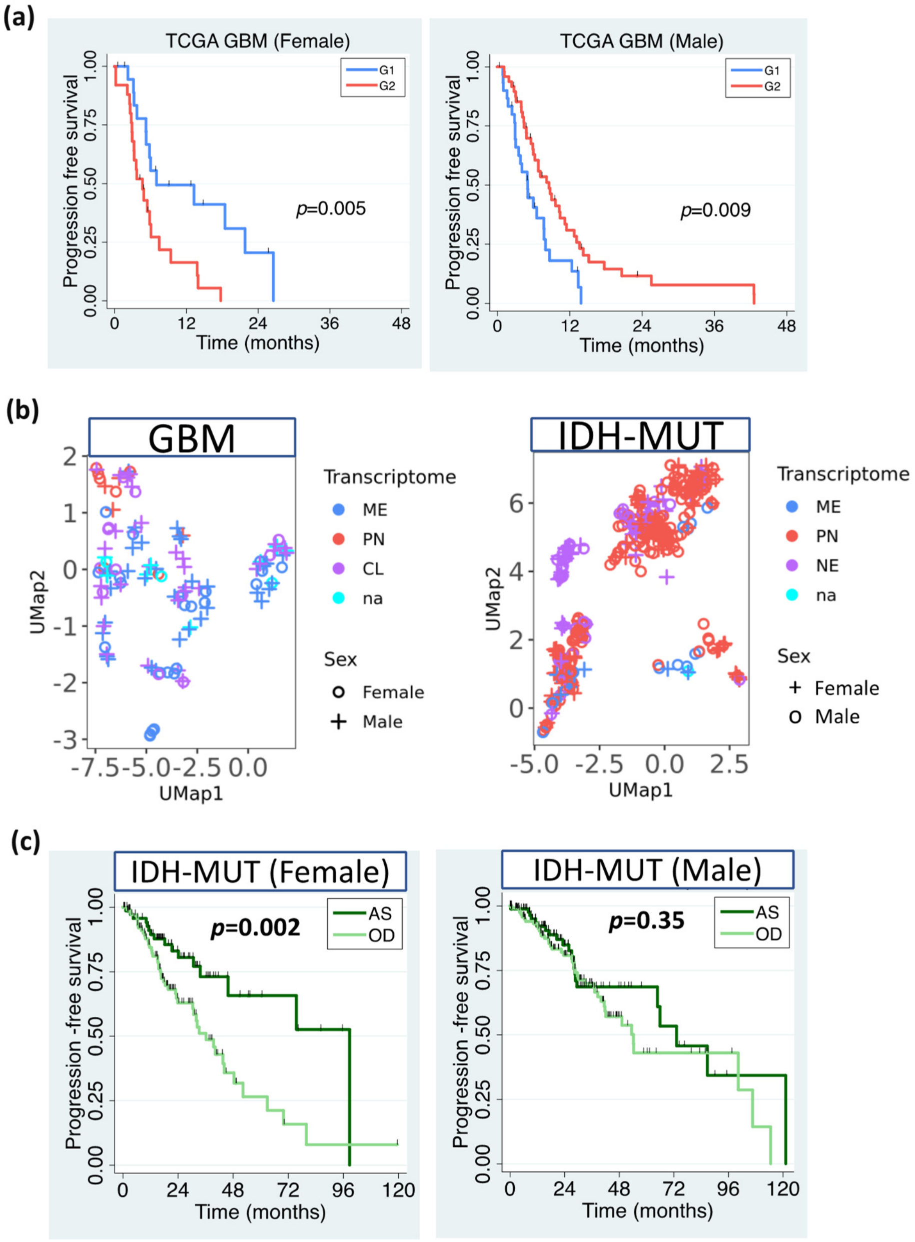

Figure S1. KM plots and UMAPs for editing-based subtyping of TCGA gliomas. (**a**) PFI of GBMs; (**b**) UMAPs of gliomas colored based on transcriptome subtypes; (**c**) PFI of IDH-MUT tumors (based on AS and OD classification).

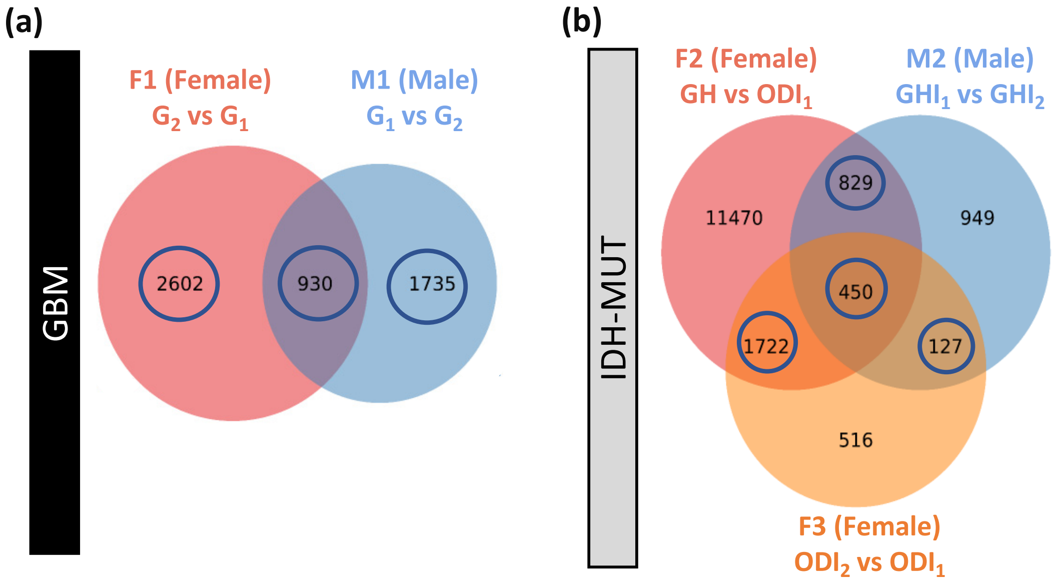

Figure S2. Differentially expressed genes between high-risk and low-risk gliomas in TCGA. (**a**) GBM (F1 and M1) and (**b**) IDH-MUT tumors (F2, F3 and M2). Comparisons are made for GBM (F1: G_2 vs. G_1 and M1: G_1 vs. G_2) and IDH-MUT gliomas (F2: GH vs. ODI_1; F3: ODI_2 vs. ODI_1 and M2: GHI_1 vs. GHI_2).

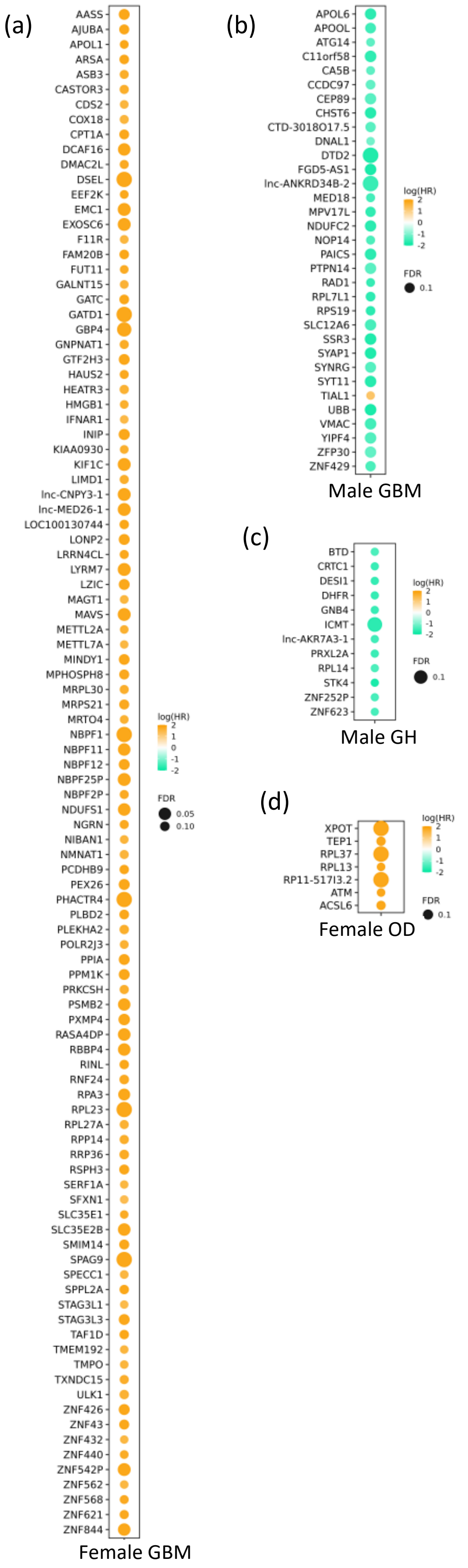

Figure S3. Age-adjusted hazard ratios of prognostic genes in TCGA gliomas. (**a**) Female GBM; (**b**)Male GBM; (**c**) Male GH and (**d**) Female OD.

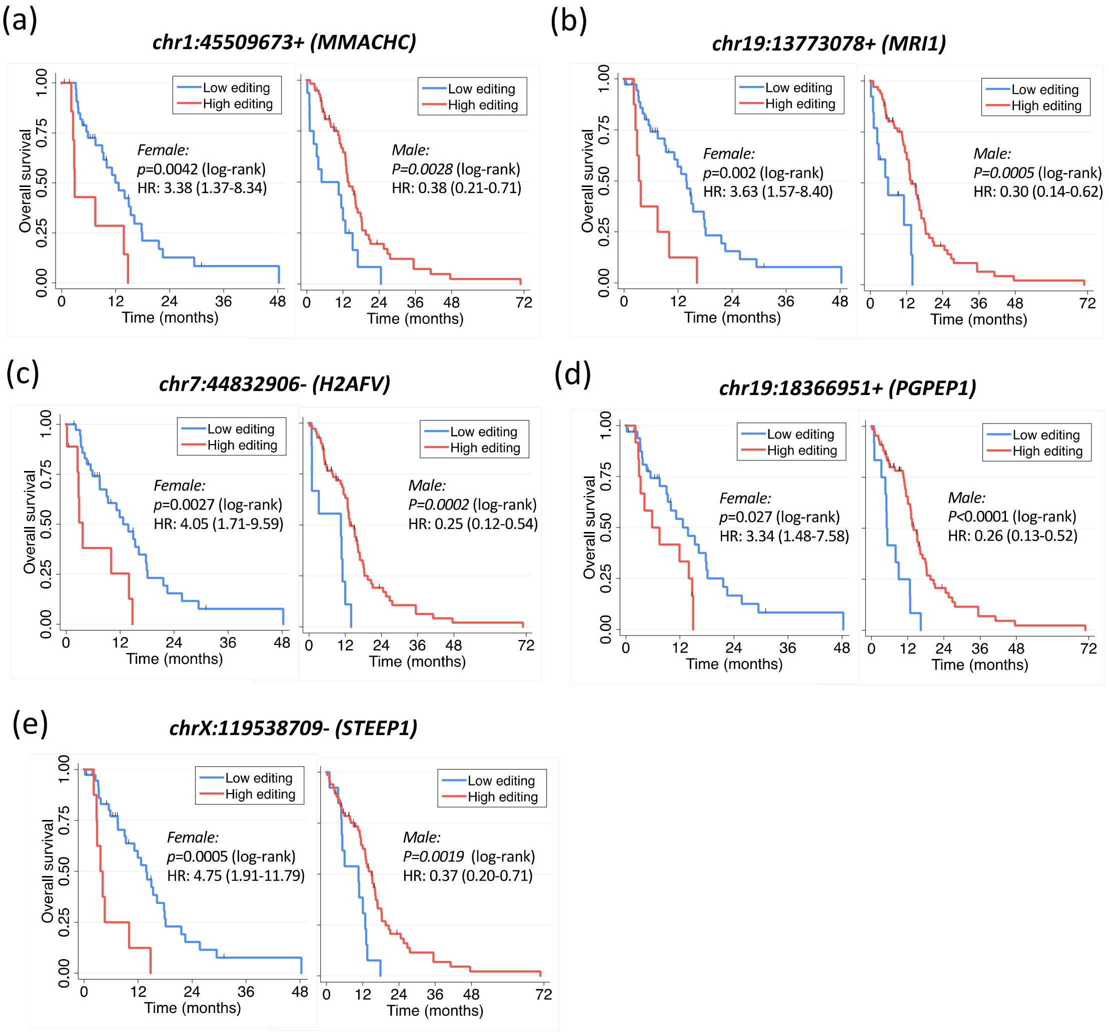

Figure S4. Kaplan-Meier curves for prognostic DESs shared by TCGA female and male GBM tumors. (**a**) chr1:45509673+ (MMACHC), (**b**) chr19:13773078+ (MRI1), (**c**) chr7:44832906- (H2AFV), (**d**) chr19:18366951+ (PGPEP1) and (**e**) chrX:119538709- (STEEP1). High vs. low: top 30% vs. bottom 70% for females and top 70% vs. bottom 30% for males.

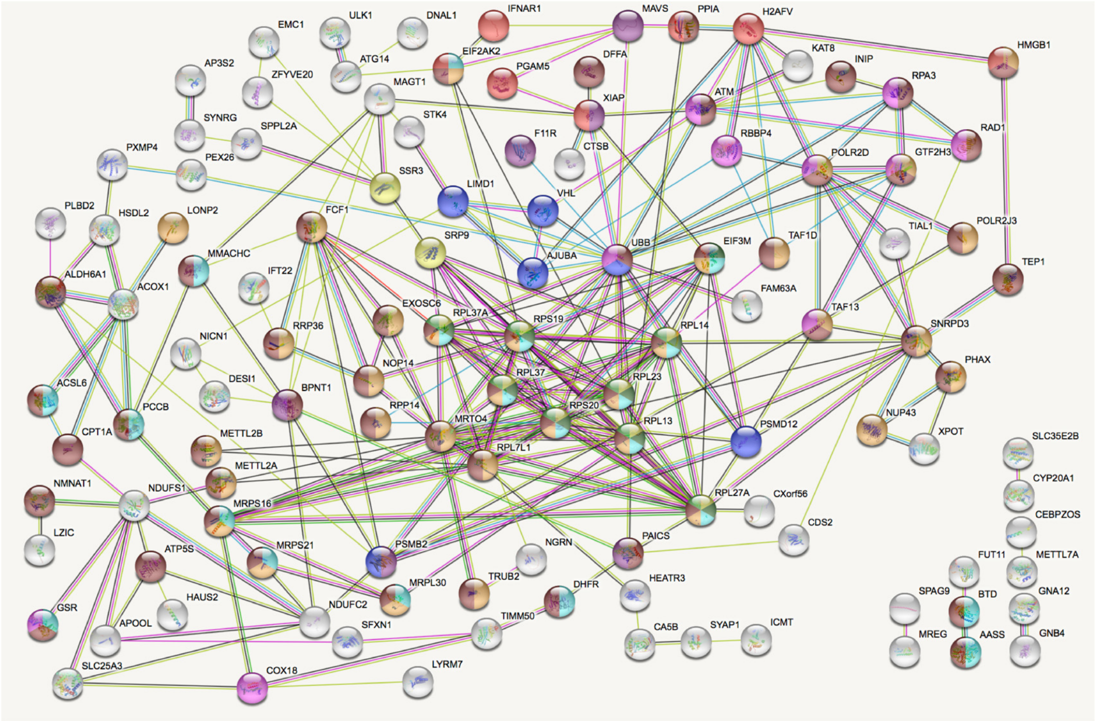

GO-term	Biological process	FDR
GO:0006614	SRP-dependent cotranslational protein targeting to membrane	1.1e-6
GO:0010467	gene expression	4.9e-05
GO:0043603	cellular amide metabolic process	2.8e-05
GO:0034641	cellular nitrogen compound metabolic process	5.0e-08

number of nodes: 190
number of edges: 260
average node degree: 2.74
avg. local clustering coefficient: 0.315
expected number of edges: 154
PPI enrichment p-value: 3.66e-15

ID	KEGG pathway	FDR
hsa04217	Necroptosis	0.0093

ID	Reactome pathway	FDR
HSA-1234176	Oxygen-dependent proline hydroxylation of Hypoxia-inducible Factor Alpha	0.0019
HSA-192823	Viral mRNA Translation	8.8e-05
HSA-3700989	Transcriptional Regulation by TP53	0.04

Figure S5. PPI network of 190 prognostic genes and selected pathways from ORA analysis of the 117 prognostic genes that were connected.

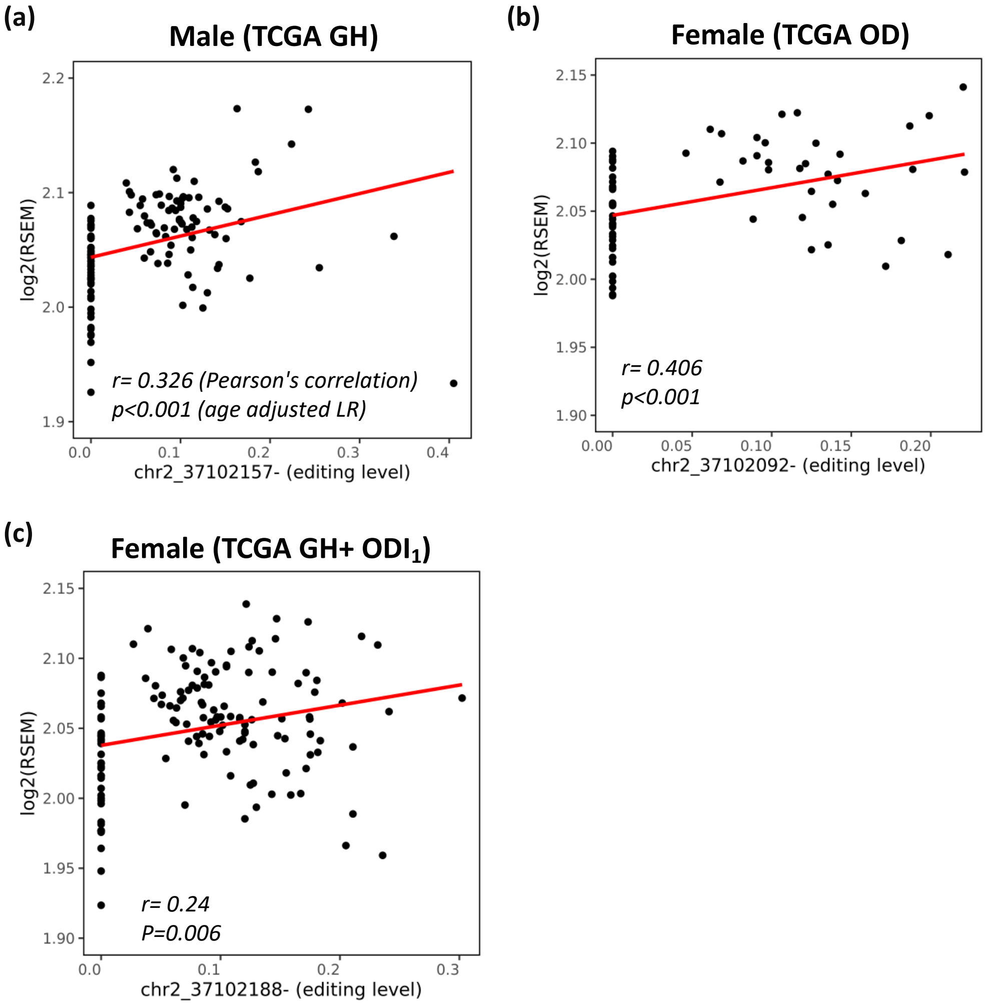

Figure S6. Editing-expression correlations of EIF2AK2 in TGGA IDH-MUT tumors. (**a**) Male GH; (**b**) Female OD; (**c**) Female GH and ODI_1.

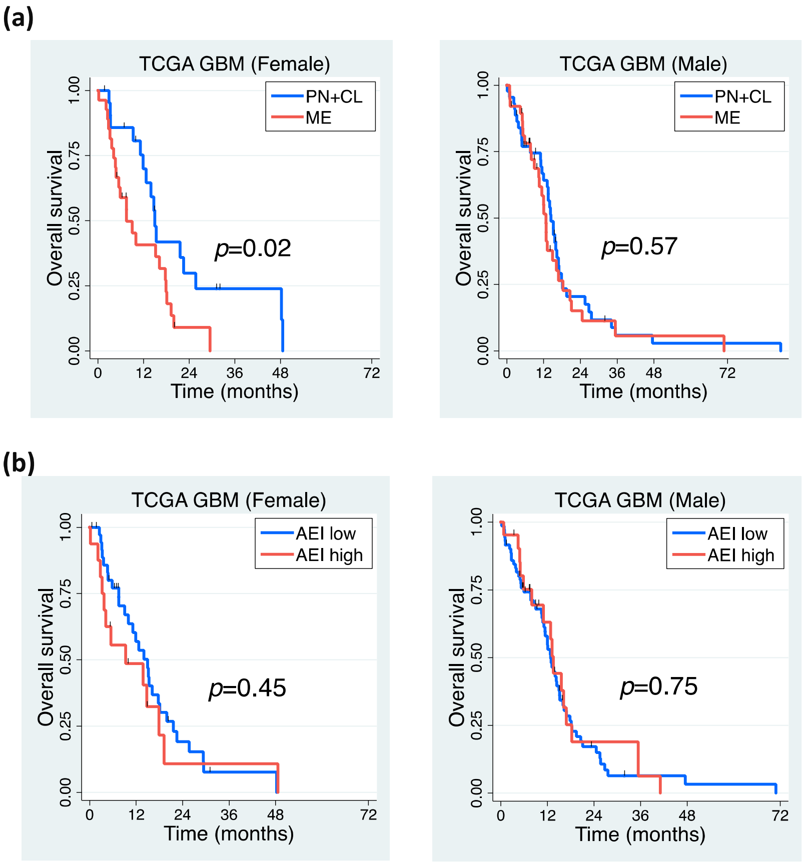

Figure S7. KM plots for TCGA GBM samples based on (**a**) transcriptome-based subtyping and (**b**) Alu editing index (AEI). PN: proneural; CL: classical; ME: mesenchymal. AEI values and cutoffs were obtained from Silvestris et al. [1]. AEI high: AEI >0.008 for females and >0.0078 for males.

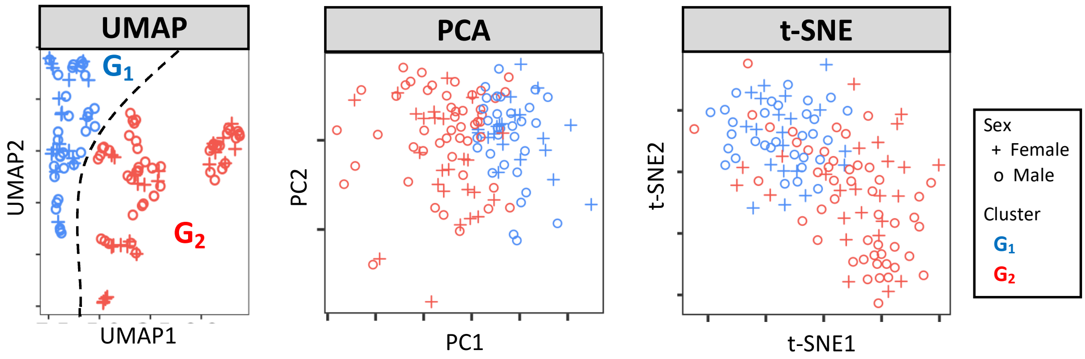

Figure S8. Visualization of TCGA GBM samples using three dimension reduction techniques (UMAP, PCA and t-SNE). Samples were colored based on UMAP-based subtyping.

Table S1. Confusion matrix and performance measures of random forest (RF) models that predict mutation status of IDH and 1p/19q in TCGA samples with 10-fold cross validation.

RF Model	Label	Prediction		Sensitivity	Specificity	F1 score	AUC
**IDHwt vs.**		IDHwt	IDHmut	0.998	0.998	0.998	1.0
**IDHmut**	IDHwt	421	1				
	IDHmu	1	446				
**RF Model**	**Label**	**Prediction**		**Sensitivity**	**Specificity**	**F1 score**	**AUC**
**noncodel**		noncodel	codel	0.989	0.994	0.993	0.999
**vs. codel**	noncodel	272	3				
	codel	1	171				

Table S2. Hazard Ratios (HRs) and 95% confidence intervals (CIs) of covariates for overall survival of GBM patients using Cox regression.

	Factor	HR (95% CIs)	*p* value
**Females**			
	Age	1.06 (1.00 - 1.12)	0.047
	Cluster (G_2)	4.40 (1.09 - 17.82)	0.038
	MGMT (yes)	0.35 (0.14 - 0.86)	0.022
	TERT (yes)	1.65 (0.40 - 6.77)	0.486
	Expression subtype		
	Proneural	Ref	
	Classical	0.91 (0.250 - 3.31)	0.885
	Mesenchymal	1.32 (0.266 - 6.50)	0.737
**Males**			
	Age	1.02 (0.98 - 1.06)	0.301
	Cluster (G_2)	0.36 (0.17 - 0.76)	0.008
	MGMT (yes)	1.03 (0.49 - 2.15)	0.942
	TERT (yes)	1.21 (0.30 - 4.94)	0.786
	Expression subtype		
	Proneural	Ref	
	Classical	0.04 (0.00 - 0.74)	0.031
	Mesenchymal	0.08 (0.00 - 1.44)	0.086

Table S3. IPA canonical pathway analysis on genes with differential editing (DE)and those without editing (nonDE).

Canonical Pathways	nonDE					DE				
	F1	F2	F3	M1	M2	F1	F2	F3	M1	M2
EIF2 Signaling	0.23	0.14	0	0.22	0	1.23	1.73	3.52	1.72	2.88
Assembly of RNA Polymerase II Complex	0.13	0.52	0.05	0.1	0.03	1.05	1.3	2.55	1.44	2.88
Oleate Biosynthesis II (Animals)	0.13	0.1	0.05	0.1	0.16	1.06	2.04	1	1.72	1.87
Induction of Apoptosis by HIV1	0	0.05	0	0	0	1.53	1.09	1.1	1.46	0.95
Role of PKR in Interferon Induction and Antiviral Response	0	0.05	0	0	0	1.23	0.99	0.97	1.7	0.95
Nucleotide Excision Repair Pathway	0.13	0.12	0.04	0.02	0.04	0.98	1.09	0.97	1.4	0.86
Granzyme B Signaling	0	0.08	0	0	0	1.53	0.39	0.97	1.59	0.95
Lysine Degradation II	0	0	0	0	0	0.99	1.09	0.97	0.94	0.95
Tumoricidal Function of Hepatic Natural Killer Cells	0	0.05	0	0	0	1.23	0.38	0.72	1.72	0.62
Death Receptor Signaling	0.12	0	0.01	0.08	0.04	0.78	1.09	0.5	1.37	0.6
MYC Mediated Apoptosis Signaling	0	0.25	0.01	0	0.03	1.05	0.12	0.66	1.44	0.95
NER (Nucleotide Excision Repair, Enhanced Pathway)	0.13	0.11	0.05	0.13	0.21	0.66	1.3	0.48	0.8	0.29
Retinoic acid Mediated Apoptosis Signaling	0	0	0	0	0	0.94	0.61	0.53	1.24	0.8
Aryl Hydrocarbon Receptor Signaling	0.12	0.52	0.05	0.22	0.21	1.02	0.11	0.82	0.41	0.57
Purine Nucleotides De Novo Biosynthesis II	0.13	0	0.01	0.22	0.16	0.56	1.3	0.54	0.42	0.5
TWEAK Signaling	0	0.03	0	0	0	0.94	0.24	0.5	1.37	0.35
Assembly of RNA Polymerase I Complex	0	0.25	0.01	0	0.16	0.53	0.12	0.53	1.24	0.44
Antigen Presentation Pathway	0.83	0.39	0.05	1.28	0.5	0	0	0	0	0
Glutamate Receptor Signaling	0	0.05	0.01	0.04	0.1	0.18	1.09	0.82	0.05	0.65

Values represents $\log_{10}$(false discovery rate)

Table S4. Gene ontology analysis of genes with differential editing (DE)and those without editing (nonDE).

Category	#term ID	term description	false discovery rate
F1_DE	GO:0006605	protein targeting	0.0065
	GO:0006886	intracellular protein transport	0.0065
	GO:0008152	metabolic process	0.0065
	GO:0009059	macromolecule biosynthetic process	0.0065
	GO:0010467	gene expression	0.0065
	GO:0015031	protein transport	0.0065
	GO:0034641	cellular nitrogen compound metabolic process	0.0065
	GO:0034645	cellular macromolecule biosynthetic process	0.0065
	GO:0042886	amide transport	0.0065
	GO:0044237	cellular metabolic process	0.0065
	GO:0045184	establishment of protein localization	0.0065
	GO:0046907	intracellular transport	0.0065
	GO:0051649	establishment of localization in cell	0.0068
	GO:0071705	nitrogen compound transport	0.007
	GO:0006139	nucleobase-containing compound metabolic process	0.0133
	GO:0070727	cellular macromolecule localization	0.0142
	GO:0008104	protein localization	0.0147
	GO:0034470	ncRNA processing	0.0147
	GO:0046483	heterocycle metabolic process	0.015
	GO:0051641	cellular localization	0.016
	GO:0034613	cellular protein localization	0.0187
	GO:0006612	protein targeting to membrane	0.0189
	GO:0006807	nitrogen compound metabolic process	0.0194
	GO:0016070	RNA metabolic process	0.0197
	GO:0006725	cellular aromatic compound metabolic process	0.0202
	GO:0044249	cellular biosynthetic process	0.0206
	GO:0090150	establishment of protein localization to membrane	0.0209
	GO:0072594	establishment of protein localization to organelle	0.0221
	GO:0033036	macromolecule localization	0.0236
	GO:0034660	ncRNA metabolic process	0.0245
	GO:0045047	protein targeting to ER	0.0249
	GO:0071702	organic substance transport	0.0249
	GO:0090304	nucleic acid metabolic process	0.0252
	GO:0044238	primary metabolic process	0.0265

	GO:0006260	DNA replication	0.0307
	GO:0022613	ribonucleoprotein complex biogenesis	0.0322
	GO:0070972	protein localization to endoplasmic reticulum	0.0384
	GO:0016032	viral process	0.0413
	GO:0009058	biosynthetic process	0.0481
F2_DE	GO:0034641	cellular nitrogen compound metabolic process	0.0012
	GO:0006605	protein targeting	0.0083
	GO:0008152	metabolic process	0.0083
	GO:0044237	cellular metabolic process	0.0083
	GO:0046907	intracellular transport	0.0083
	GO:0006807	nitrogen compound metabolic process	0.0138
	GO:0046483	heterocycle metabolic process	0.0138
	GO:0043603	cellular amide metabolic process	0.0144
	GO:0044238	primary metabolic process	0.0144
	GO:0006725	cellular aromatic compound metabolic process	0.016
	GO:0006139	nucleobase-containing compound metabolic process	0.0176
	GO:0010467	gene expression	0.0291
	GO:0015031	protein transport	0.0291
	GO:0044271	cellular nitrogen compound biosynthetic process	0.0291
	GO:0051649	establishment of localization in cell	0.0291
	GO:1901360	organic cyclic compound metabolic process	0.0298
	GO:0006886	intracellular protein transport	0.0331
	GO:0019080	viral gene expression	0.0331
	GO:0045047	protein targeting to ER	0.0331
	GO:0071704	organic substance metabolic process	0.0331
	GO:0072594	establishment of protein localization to organelle	0.0331
	GO:0006612	protein targeting to membrane	0.0344
	GO:0016032	viral process	0.0344
	GO:0019083	viral transcription	0.0344
	GO:0051641	cellular localization	0.0344
	GO:0034645	cellular macromolecule biosynthetic process	0.0405
	GO:0044249	cellular biosynthetic process	0.0405
	GO:0006614	SRP-dependent cotranslational protein targeting to membrane	0.0413
F3_DE	GO:0034641	cellular nitrogen compound metabolic process	0.00049
	GO:0006605	protein targeting	0.00062
	GO:0044237	cellular metabolic process	0.00062
	GO:0045047	protein targeting to ER	0.00062
	GO:0016032	viral process	0.00072

GO:0070972	protein localization to endoplasmic reticulum	0.00072
GO:0006612	protein targeting to membrane	0.00075
GO:0010467	gene expression	0.00099
GO:0034645	cellular macromolecule biosynthetic process	0.00099
GO:0044403	symbiotic process	0.00099
GO:0090150	establishment of protein localization to membrane	0.00099
GO:0009059	macromolecule biosynthetic process	0.001
GO:0006614	SRP-dependent cotranslational protein targeting to membrane	0.0011
GO:0006807	nitrogen compound metabolic process	0.0011
GO:0008152	metabolic process	0.0011
GO:0044249	cellular biosynthetic process	0.0014
GO:0044271	cellular nitrogen compound biosynthetic process	0.0014
GO:0046907	intracellular transport	0.0014
GO:0072594	establishment of protein localization to organelle	0.0018
GO:0044238	primary metabolic process	0.0019
GO:0006886	intracellular protein transport	0.0021
GO:0016070	RNA metabolic process	0.0034
GO:1901576	organic substance biosynthetic process	0.0034
GO:0009058	biosynthetic process	0.0038
GO:0015031	protein transport	0.0042
GO:0045184	establishment of protein localization	0.0042
GO:0006139	nucleobase-containing compound metabolic process	0.0062
GO:0043603	cellular amide metabolic process	0.0062
GO:0046483	heterocycle metabolic process	0.0065
GO:0051649	establishment of localization in cell	0.0065
GO:0019080	viral gene expression	0.0094
GO:0071704	organic substance metabolic process	0.0094
GO:0006725	cellular aromatic compound metabolic process	0.0097
GO:0019083	viral transcription	0.0122
GO:0006413	translational initiation	0.0151
GO:1901360	organic cyclic compound metabolic process	0.0182
GO:0006412	translation	0.0201
GO:0090304	nucleic acid metabolic process	0.0201
GO:0072657	protein localization to membrane	0.0213
GO:0051641	cellular localization	0.0224
GO:0071705	nitrogen compound transport	0.0249
GO:0033365	protein localization to organelle	0.0258
GO:1901575	organic substance catabolic process	0.0274

	GO:0043604	amide biosynthetic process	0.0315
	GO:0034613	cellular protein localization	0.0373
	GO:0042795	snRNA transcription by RNA polymerase II	0.0398
	GO:0009056	catabolic process	0.0433
	GO:0098781	ncRNA transcription	0.0485
	GO:0008104	protein localization	0.049
M1_DE	GO:0034641	cellular nitrogen compound metabolic process	2.63E-05
	GO:0010467	gene expression	7.48E-05
	GO:0006605	protein targeting	0.00043
	GO:0016070	RNA metabolic process	0.00047
	GO:0006139	nucleobase-containing compound metabolic process	0.00065
	GO:0044237	cellular metabolic process	0.00065
	GO:0006725	cellular aromatic compound metabolic process	0.00077
	GO:0008152	metabolic process	0.00077
	GO:0009059	macromolecule biosynthetic process	0.00077
	GO:0046483	heterocycle metabolic process	0.00077
	GO:0034645	cellular macromolecule biosynthetic process	0.00083
	GO:0090304	nucleic acid metabolic process	0.0012
	GO:0006612	protein targeting to membrane	0.0015
	GO:0044249	cellular biosynthetic process	0.0018
	GO:0046907	intracellular transport	0.0018
	GO:0009058	biosynthetic process	0.0021
	GO:1901360	organic cyclic compound metabolic process	0.0023
	GO:0045047	protein targeting to ER	0.0034
	GO:0006807	nitrogen compound metabolic process	0.0035
	GO:0006886	intracellular protein transport	0.0035
	GO:1901576	organic substance biosynthetic process	0.0035
	GO:0006613	cotranslational protein targeting to membrane	0.0044
	GO:0044238	primary metabolic process	0.0044
	GO:0090150	establishment of protein localization to membrane	0.0044
	GO:0034470	ncRNA processing	0.0048
	GO:0015031	protein transport	0.0057
	GO:0044271	cellular nitrogen compound biosynthetic process	0.0063
	GO:0070972	protein localization to endoplasmic reticulum	0.0065
	GO:0045184	establishment of protein localization	0.0075
	GO:0006396	RNA processing	0.0077
	GO:0034660	ncRNA metabolic process	0.0077
	GO:0071705	nitrogen compound transport	0.0093

	GO:0042886	amide transport	0.0094
	GO:0006614	SRP-dependent cotranslational protein targeting to membrane	0.01
	GO:0019080	viral gene expression	0.011
	GO:0071704	organic substance metabolic process	0.011
	GO:0072594	establishment of protein localization to organelle	0.011
	GO:0006412	translation	0.0121
	GO:0043603	cellular amide metabolic process	0.0127
	GO:0016032	viral process	0.0134
	GO:0071702	organic substance transport	0.0388
	GO:0019083	viral transcription	0.0389
	GO:0022613	ribonucleoprotein complex biogenesis	0.0389
	GO:0006518	peptide metabolic process	0.0434
	GO:0043170	macromolecule metabolic process	0.0478
M2_DE	GO:0034641	cellular nitrogen compound metabolic process	4.37E-07
	GO:0009059	macromolecule biosynthetic process	5.08E-06
	GO:0034645	cellular macromolecule biosynthetic process	5.08E-06
	GO:0006139	nucleobase-containing compound metabolic process	1.56E-05
	GO:0046483	heterocycle metabolic process	1.72E-05
	GO:0044249	cellular biosynthetic process	2.92E-05
	GO:0006605	protein targeting	3.01E-05
	GO:1901576	organic substance biosynthetic process	3.51E-05
	GO:0006725	cellular aromatic compound metabolic process	4.09E-05
	GO:0009058	biosynthetic process	4.14E-05
	GO:0044237	cellular metabolic process	4.14E-05
	GO:0044271	cellular nitrogen compound biosynthetic process	4.14E-05
	GO:0046907	intracellular transport	4.14E-05
	GO:0010467	gene expression	4.37E-05
	GO:0016070	RNA metabolic process	7.08E-05
	GO:0090304	nucleic acid metabolic process	7.08E-05
	GO:1901360	organic cyclic compound metabolic process	0.00013
	GO:0016032	viral process	0.00014
	GO:0008152	metabolic process	0.0002
	GO:0045047	protein targeting to ER	0.00023
	GO:0006807	nitrogen compound metabolic process	0.00026
	GO:0043603	cellular amide metabolic process	0.00026
	GO:0006886	intracellular protein transport	0.00027
	GO:0044403	symbiotic process	0.0003
	GO:0015031	protein transport	0.00033

	GO:0044238	primary metabolic process	0.00036
	GO:0045184	establishment of protein localization	0.00045
	GO:0070972	protein localization to endoplasmic reticulum	0.00054
	GO:0006612	protein targeting to membrane	0.00087
	GO:0090150	establishment of protein localization to membrane	0.00087
	GO:0006614	SRP-dependent cotranslational protein targeting to membrane	0.00093
	GO:0072594	establishment of protein localization to organelle	0.00096
	GO:0071704	organic substance metabolic process	0.0013
	GO:0071705	nitrogen compound transport	0.0014
	GO:0051649	establishment of localization in cell	0.0035
	GO:0043604	amide biosynthetic process	0.0066
	GO:0006412	translation	0.0086
	GO:0051641	cellular localization	0.0095
	GO:0006518	peptide metabolic process	0.0111
	GO:0070727	cellular macromolecule localization	0.0111
	GO:0019080	viral gene expression	0.0156
	GO:0034613	cellular protein localization	0.0168
	GO:0019083	viral transcription	0.0176
	GO:0044260	cellular macromolecule metabolic process	0.0176
	GO:0043170	macromolecule metabolic process	0.0179
	GO:0072657	protein localization to membrane	0.0179
	GO:0034654	nucleobase-containing compound biosynthetic process	0.0218
	GO:0008104	protein localization	0.0227
	GO:0071702	organic substance transport	0.0227
	GO:0006413	translational initiation	0.0234
	GO:1901566	organonitrogen compound biosynthetic process	0.0234
	GO:0033365	protein localization to organelle	0.0305
	GO:0042795	snRNA transcription by RNA polymerase II	0.0329
	GO:0006402	mRNA catabolic process	0.0353
	GO:0000956	nuclear-transcribed mRNA catabolic process	0.0392
	GO:0018130	heterocycle biosynthetic process	0.0392
	GO:0019438	aromatic compound biosynthetic process	0.0461
	GO:0016071	mRNA metabolic process	0.0488
F1_nonDE	GO:0034641	cellular nitrogen compound metabolic process	0.00015
F2_nonDE	GO:0016070	RNA metabolic process	0.00018
	GO:0034641	cellular nitrogen compound metabolic process	0.00018
	GO:0090304	nucleic acid metabolic process	0.00058

	GO:0006139	nucleobase-containing compound metabolic process	0.0043
	GO:0006396	RNA processing	0.0043
	GO:0010467	gene expression	0.0043
	GO:0044237	cellular metabolic process	0.0077
	GO:1901796	regulation of signal transduction by p53 class mediator	0.0165
	GO:0046483	heterocycle metabolic process	0.0172
	GO:0006725	cellular aromatic compound metabolic process	0.0184
F3_nonDE	GO:0034641	cellular nitrogen compound metabolic process	0.00074
	GO:0006139	nucleobase-containing compound metabolic process	0.0108
	GO:0090304	nucleic acid metabolic process	0.0127
	GO:0006725	cellular aromatic compound metabolic process	0.0209
	GO:0016070	RNA metabolic process	0.0209
	GO:0046483	heterocycle metabolic process	0.0209
	GO:0044237	cellular metabolic process	0.0494
	GO:0010467	gene expression	0.0495

Table S5. Cancer-associated functions of genes with coding DES in Figure 4d.

Gene	PMID	Title
ANKDD1A	30082910	Hypermethylated gene ANKDD1A is a candidate tumor suppressor that interacts with FIH1 and decreases HIF1α stability to inhibit cell autophagy in the glioblastoma multiforme hypoxia microenvironment
AZIN1	27870265	Antizyme inhibitor 1: a potential carcinogenic molecule
CADPS	21798848	Genome-wide molecular characterization of central nervous system primitive neuroectodermal tumor and pineoblastoma
FLNB	23717429	Development of Biomarkers for Screening Hepatocellular Carcinoma Using Global Data Mining and Multiple Reaction Monitoring
BEST1	19470678	Bestrophin 1 promotes epithelial-to-mesenchymal transition of renal collecting duct cells
GRIA2	22644307	Identification of differentially expressed genes according to chemosensitivity in advanced ovarian serous adenocarcinomas: expression of GRIA2 predicts better survival
GRIA3	20689760	Glutamate Receptor GRIA3-Target of CUX1 and Mediator of Tumor Progression in Pancreatic Cancer
Nicastrin (NCSTN)	32631394	NCSTN promotes hepatocellular carcinoma cell growth and metastasis via β-catenin activation in a Notch1/AKT dependent manner
NOP14	22425761	NOP14 promotes proliferation and metastasis of pancreatic cancer cells.
GRIK2	19824040	Glutamate receptor, ionotropic, kainate 2 silencing by DNA hypermethylation possesses tumor suppressor function in gastric cancer
SH3BP2	29885053	Silencing of adaptor protein SH3BP2 reduces KIT/PDGFRA receptors expression and impairs gastrointestinal stromal tumors growth
SRP9	18549262	Proteomic expression analysis of surgical human colorectal cancer tissues: up-regulation of PSB7, PRDX1, and SRP9 and hypoxic adaptation in cancer

Table S6. List of editing-regulated and/or prognostic genes in TCGA gliomas.

ENSEMBL	refGene	Func.refGene	Status
ENSG00000004468	CD38	UTR3	Editing-regulated
ENSG00000008294	SPAG9	UTR3	prognostic
ENSG00000008311	AASS	UTR3	prognostic
ENSG00000008988	RPS20	UTR3	prognostic
ENSG00000018610	CXorf56	UTR3	prognostic
ENSG00000023228	NDUFS1	UTR3	prognostic
ENSG00000037757	MRI1	UTR3	Both
ENSG00000049239	H6PD	UTR3	Editing-regulated
ENSG00000050393	MCUR1	UTR3	Editing-regulated
ENSG00000053372	MRTO4	UTR3	prognostic
ENSG00000055332	EIF2AK2	UTR3	Both
ENSG00000068489	PRR11	UTR3	Editing-regulated
ENSG00000068654	POLR1A	UTR3	Editing-regulated
ENSG00000072071	ADGRL1	UTR3	Editing-regulated
ENSG00000075415	SLC25A3	UTR3	Both
ENSG00000082701	GSK3B	UTR3	Editing-regulated
ENSG00000084112	SSH1	UTR3	Editing-regulated
ENSG00000087266	SH3BP2	UTR3	Editing-regulated
ENSG00000087269	NOP14	exonic	prognostic
ENSG00000087995	METTL2A	UTR3	prognostic
ENSG00000088888	MAVS	UTR3	Both
ENSG00000089050	RBBP9	UTR3	prognostic
ENSG00000100028	SNRPD3	UTR3	Both
ENSG00000100228	RAB36	UTR3	Editing-regulated
ENSG00000100299	ARSA	UTR3	prognostic
ENSG00000100342	APOL1	UTR3	Both
ENSG00000100350	FOXRED2	UTR3	prognostic
ENSG00000100364	KIAA0930	UTR3	Both
ENSG00000100418	DESI1	UTR3	prognostic
ENSG00000100522	GNPNAT1	UTR3	Both
ENSG00000101109	STK4	UTR3	prognostic
ENSG00000101190	TCFL5	UTR3	Editing-regulated
ENSG00000101236	RNF24	UTR3	prognostic
ENSG00000101290	CDS2	UTR3	prognostic
ENSG00000101347	SAMHD1	UTR3	Editing-regulated

ENSG00000101417	PXMP4	UTR3	prognostic
ENSG00000101966	XIAP	UTR3	Both
ENSG00000102158	MAGT1	UTR3	Both
ENSG00000102908	NFAT5	UTR3	Editing-regulated
ENSG00000102910	LONP2	UTR3	Both
ENSG00000103319	EEF2K	UTR3	prognostic
ENSG00000103510	KAT8	intronic	prognostic
ENSG00000104687	GSR	UTR3	prognostic
ENSG00000104738	MCM4	UTR3	Editing-regulated
ENSG00000104805	NUCB1	downstream	Editing-regulated
ENSG00000105197	TIMM50	UTR3	Both
ENSG00000105372	RPS19	UTR3	prognostic
ENSG00000105662	CRTC1	UTR3	Both
ENSG00000105968	H2AFV	intronic	Both
ENSG00000106399	RPA3	downstream	prognostic
ENSG00000106546	AHR	UTR3	prognostic
ENSG00000110090	CPT1A	UTR3	prognostic
ENSG00000110696	C11orf58	UTR3	prognostic
ENSG00000111358	GTF2H3	UTR3	Both
ENSG00000113456	RAD1	UTR3	Both
ENSG00000113621	TXNDC15	UTR3	Both
ENSG00000114054	PCCB	intronic	Both
ENSG00000114450	GNB4	UTR3	Both
ENSG00000114850	SSR3	UTR3	prognostic
ENSG00000115239	ASB3	intronic	prognostic
ENSG00000116199	FAM20B	UTR3	Both
ENSG00000116237	ICMT	UTR3	prognostic
ENSG00000117899	MESD	UTR3	Editing-regulated
ENSG00000118242	MREG	UTR3	prognostic
ENSG00000119004	CYP20A1	UTR3	prognostic
ENSG00000119471	HSDL2	UTR3	prognostic
ENSG00000119616	FCF1	UTR3	Both
ENSG00000119661	DNAL1	UTR3	prognostic
ENSG00000119711	ALDH6A1	UTR3	Both
ENSG00000119820	YIPF4	UTR3	Both
ENSG00000120253	NUP43	UTR3	Both
ENSG00000120784	ZFP30	UTR3	prognostic
ENSG00000120802	TMPO	UTR3	Both

ENSG00000121289	CEP89	UTR3	prognostic
ENSG00000122378	PRXL2A	UTR3	Both
ENSG00000124541	RRP36	intronic	prognostic
ENSG00000125375	DMAC2L	UTR3	prognostic
ENSG00000125691	RPL23	UTR3	prognostic
ENSG00000125741	OPA3	UTR3	Editing-regulated
ENSG00000125779	PANK2	UTR3	Editing-regulated
ENSG00000126067	PSMB2	UTR3	prognostic
ENSG00000126775	ATG14	UTR3	prognostic
ENSG00000127463	EMC1	UTR3	Both
ENSG00000127526	SLC35E1	UTR3	Both
ENSG00000128050	PAICS	UTR3	Both
ENSG00000128487	SPECC1	UTR3	Both
ENSG00000128581	IFT22	UTR3	Both
ENSG00000128928	IVD	UTR3	Editing-regulated
ENSG00000129128	SPCS3	UTR3	Editing-regulated
ENSG00000129250	KIF1C	UTR3	prognostic
ENSG00000129472	RAB2B	UTR3	Editing-regulated
ENSG00000129474	AJUBA	UTR3	prognostic
ENSG00000129480	DTD2	UTR3	prognostic
ENSG00000129566	TEP1	UTR3	Both
ENSG00000130119	GNL3L	UTR3	Editing-regulated
ENSG00000130175	PRKCSH	intronic	prognostic
ENSG00000130363	RSPH3	UTR3	prognostic
ENSG00000130517	PGPEP1	UTR3	prognostic
ENSG00000130772	MED18	UTR3	prognostic
ENSG00000130818	ZNF426	UTR3	prognostic
ENSG00000131381	RBSN	UTR3	Both
ENSG00000131386	GALNT15	intronic	Both
ENSG00000132718	SYT11	UTR3	Both
ENSG00000132763	MMACHC	UTR3	prognostic
ENSG00000132849	PATJ	UTR3	Editing-regulated
ENSG00000133460	SLC2A11	UTR3	Editing-regulated
ENSG00000134086	VHL	UTR3	Both
ENSG00000134824	FADS2	intronic	Editing-regulated
ENSG00000135679	MDM2	UTR3	Editing-regulated
ENSG00000135842	NIBAN1	UTR3	prognostic
ENSG00000135956	TMEM127	UTR3	Editing-regulated

ENSG00000136816	TOR1B	exonic	Editing-regulated
ENSG00000137814	HAUS2	UTR3	Both
ENSG00000138600	SPPL2A	UTR3	prognostic
ENSG00000139178	C1RL	UTR3	Editing-regulated
ENSG00000139291	TMEM19	UTR3	prognostic
ENSG00000140199	SLC12A6	UTR3	Both
ENSG00000140749	IGSF6	UTR3	prognostic
ENSG00000141068	KSR1	UTR3	Editing-regulated
ENSG00000141569	TRIM65	UTR3	Editing-regulated
ENSG00000141905	NFIC	UTR3	Editing-regulated
ENSG00000142039	CCDC97	UTR3	prognostic
ENSG00000142166	IFNAR1	UTR3	Both
ENSG00000142751	GPN2	UTR3	Editing-regulated
ENSG00000143409	MINDY1	intronic	Both
ENSG00000143742	SRP9	exonic	prognostic
ENSG00000144231	POLR2D	UTR3	Both
ENSG00000144730	IL17RD	UTR3	Editing-regulated
ENSG00000144791	LIMD1	UTR3	Both
ENSG00000145029	NICN1	UTR3	Both
ENSG00000145545	SRD5A1	UTR3	Editing-regulated
ENSG00000145592	RPL37	UTR3	prognostic
ENSG00000145916	RMND5B	UTR3	Editing-regulated
ENSG00000146223	RPL7L1	UTR3	prognostic
ENSG00000146535	GNA12	intergenic	Both
ENSG00000147164	SNX12	downstream	Editing-regulated
ENSG00000148153	INIP	UTR3	Both
ENSG00000149100	EIF3M	UTR3	Both
ENSG00000149311	ATM	UTR3	prognostic
ENSG00000149485	FADS1	downstream	Editing-regulated
ENSG00000151176	PLBD2	UTR3	Both
ENSG00000151366	NDUFC2	UTR3	Both
ENSG00000151923	TIAL1	downstream	Both
ENSG00000152104	PTPN14	UTR3	prognostic
ENSG00000154079	SDHAF4	UTR3	Editing-regulated
ENSG00000155008	APOOL	UTR3	prognostic
ENSG00000155034	FBXL18	UTR3	Editing-regulated
ENSG00000155324	GRAMD2B	downstream	Editing-regulated
ENSG00000155393	HEATR3	UTR3	prognostic

ENSG00000155980	KIF5A	UTR3	Editing-regulated
ENSG00000156162	DPY19L4	UTR3	Both
ENSG00000156968	MPV17L	UTR3	prognostic
ENSG00000157150	TIMP4	downstream	Editing-regulated
ENSG00000157823	AP3S2	UTR3	prognostic
ENSG00000158604	TMED4	UTR3	Editing-regulated
ENSG00000158769	F11R	UTR3	Both
ENSG00000160049	DFFA	UTR3	prognostic
ENSG00000160953	PWWP3A	intronic	Editing-regulated
ENSG00000160991	ORAI2	UTR3	Editing-regulated
ENSG00000161533	ACOX1	UTR3	Both
ENSG00000162129	CLPB	UTR3	Editing-regulated
ENSG00000162441	LZIC	UTR3	prognostic
ENSG00000162521	RBBP4	UTR3	prognostic
ENSG00000162654	GBP4	UTR3	prognostic
ENSG00000162813	BPNT1	UTR3	prognostic
ENSG00000163257	DCAF16	UTR3	Both
ENSG00000163590	PPM1L	UTR3	Editing-regulated
ENSG00000163626	COX18	UTR3	Both
ENSG00000163644	PPM1K	UTR3	prognostic
ENSG00000163683	SMIM14	UTR3	Both
ENSG00000163684	RPP14	UTR3	prognostic
ENSG00000163807	KIAA1143	UTR3	prognostic
ENSG00000164398	ACSL6	UTR3	prognostic
ENSG00000164418	GRIK2	exonic	Editing-regulated
ENSG00000164466	SFXN1	UTR3	prognostic
ENSG00000164733	CTSB	UTR3	prognostic
ENSG00000164902	PHAX	UTR3	prognostic
ENSG00000165055	METTL2B	UTR3	prognostic
ENSG00000166012	TAF1D	downstream	prognostic
ENSG00000166295	ANAPC16	UTR3	Editing-regulated
ENSG00000166441	RPL27A	UTR3	prognostic
ENSG00000166822	TMEM170A	UTR3	prognostic
ENSG00000166839	ANKDD1A	exonic	Editing-regulated
ENSG00000167112	TRUB2	UTR3	prognostic
ENSG00000167526	RPL13	UTR3	prognostic
ENSG00000168255	POLR2J3	UTR3	prognostic
ENSG00000169239	CA5B	UTR3	prognostic

ENSG00000169499	PLEKHA2	UTR3	Both
ENSG00000169814	BTD	UTR3	prognostic
ENSG00000169895	SYAP1	UTR3	prognostic
ENSG00000170088	TMEM192	UTR3	prognostic
ENSG00000170315	UBB	downstream	prognostic
ENSG00000170946	DNAJC24	UTR3	Editing-regulated
ENSG00000171295	ZNF440	UTR3	prognostic
ENSG00000171451	DSEL	UTR3	prognostic
ENSG00000171466	ZNF562	UTR3	prognostic
ENSG00000171490	RSL1D1	UTR3	Editing-regulated
ENSG00000171533	MAP6	UTR3	Editing-regulated
ENSG00000172058	SERF1A	UTR3	prognostic
ENSG00000172115	CYCS	UTR3	Editing-regulated
ENSG00000172840	PDP2	UTR3	Editing-regulated
ENSG00000172888	ZNF621	UTR3	prognostic
ENSG00000173273	TNKS	UTR3	Editing-regulated
ENSG00000173614	NMNAT1	UTR3	prognostic
ENSG00000173960	UBXN2A	UTR3	Editing-regulated
ENSG00000174353	STAG3L3	ncRNA_exonic	prognostic
ENSG00000177169	ULK1	UTR3	prognostic
ENSG00000177225	GATD1	UTR3	prognostic
ENSG00000177363	LRRN4CL	UTR3	prognostic
ENSG00000177839	PCDHB9	UTR3	Both
ENSG00000179152	TCAIM	UTR3	Editing-regulated
ENSG00000181192	DHTKD1	UTR3	Editing-regulated
ENSG00000182054	IDH2	upstream;downstream	Editing-regulated
ENSG00000182180	MRPS16	UTR3	Both
ENSG00000182768	NGRN	UTR3	prognostic
ENSG00000182986	ZNF320	UTR3	Editing-regulated
ENSG00000183098	GPC6	UTR3	Editing-regulated
ENSG00000183160	TMEM119	UTR3	Editing-regulated
ENSG00000183196	CHST6	UTR3	Both
ENSG00000183309	ZNF623	UTR3	prognostic
ENSG00000184575	XPOT	UTR3	Both
ENSG00000184619	KRBA2	downstream	Editing-regulated
ENSG00000185361	TNFAIP8L1	UTR3	Editing-regulated
ENSG00000185414	MRPL30	UTR3	prognostic
ENSG00000185432	METTL7A	UTR3	Both

ENSG00000185880	TRIM69	intronic	Editing-regulated
ENSG00000186687	LYRM7	UTR3	prognostic
ENSG00000186812	ZNF397	UTR3	Editing-regulated
ENSG00000187398	LUZP2	UTR3	Editing-regulated
ENSG00000187650	VMAC	UTR3	Both
ENSG00000187994	RINL	UTR3	Both
ENSG00000188643	S100A16	downstream	Editing-regulated
ENSG00000188846	RPL14	UTR3	Both
ENSG00000188917	TRMT2B	UTR3	Editing-regulated
ENSG00000188985	DHFRP1	downstream	prognostic
ENSG00000189339	SLC35E2B	UTR3	prognostic
ENSG00000189403	HMGB1	UTR3	prognostic
ENSG00000196199	MPHOSPH8	UTR3	prognostic
ENSG00000196262	PPIA	UTR3	prognostic
ENSG00000196743	GM2A	UTR3	Editing-regulated
ENSG00000196922	ZNF252P	ncRNA_exonic	Both
ENSG00000196968	FUT11	downstream	prognostic
ENSG00000197013	ZNF429	intronic	prognostic
ENSG00000197162	ZNF785	UTR3	Editing-regulated
ENSG00000197170	PSMD12	UTR3	prognostic
ENSG00000197429	IPP	intronic	Both
ENSG00000197756	RPL37A	UTR3	Both
ENSG00000197780	TAF13	intronic	prognostic
ENSG00000198453	ZNF568	UTR3	prognostic
ENSG00000198521	ZNF43	UTR3	prognostic
ENSG00000203705	TATDN3	UTR3	Editing-regulated
ENSG00000204138	PHACTR4	UTR3	prognostic
ENSG00000205583	STAG3L1	downstream	prognostic
ENSG00000211448	DIO2	UTR3	Editing-regulated
ENSG00000213853	EMP2	UTR3	Editing-regulated
ENSG00000214021	TTLL3	UTR3	Editing-regulated
ENSG00000215193	PEX26	UTR3	prognostic
ENSG00000218739	CEBPZOS	UTR3	prognostic
ENSG00000219481	NBPF1	UTR3	prognostic
ENSG00000221963	APOL6	UTR3	prognostic
ENSG00000223496	EXOSC6	UTR3	prognostic
ENSG00000223547	ZNF844	UTR3	prognostic
ENSG00000225733	FGD5-AS1	ncRNA_exonic	prognostic

ENSG00000227001	NBPF2P	downstream	prognostic
ENSG00000228716	DHFR	UTR3	prognostic
ENSG00000231113	lnc-CNPY3-1	ncRNA_exonic	prognostic
ENSG00000233297	RASA4DP	ncRNA_exonic	prognostic
ENSG00000239521	CASTOR3	intronic	prognostic
ENSG00000240225	ZNF542P	ncRNA_exonic	prognostic
ENSG00000241258	CRCP	UTR3	Editing-regulated
ENSG00000242498	ARPIN	UTR3	Editing-regulated
ENSG00000247077	PGAM5	UTR3	Both
ENSG00000249042	lnc-ANKRD34B-2	ncRNA_exonic	prognostic
ENSG00000256087	ZNF432	UTR3	prognostic
ENSG00000257218	GATC	UTR3	prognostic
ENSG00000263956	NBPF11	UTR3	prognostic
ENSG00000266472	MRPS21	UTR3	prognostic
ENSG00000268043	NBPF12	UTR3	prognostic
ENSG00000268205	lnc-ZNF460-2	ncRNA_exonic	prognostic
ENSG00000269044	lnc-MED26-1	ncRNA_exonic	prognostic
ENSG00000271605	MILR1	UTR3	Editing-regulated
ENSG00000272150	NBPF25P	ncRNA_intronic	prognostic
ENSG00000273270	lnc-IMPDH1-3	ncRNA_exonic	prognostic
ENSG00000275066	SYNRG	UTR3	prognostic
ENSG00000277977	CTD-3018O17.5	downstream	prognostic
ENSG00000279118	RP11-517I3.2	ncRNA_exonic	prognostic
LOC100130744	LOC100130744	ncRNA_intronic	prognostic
LOC100506730	lnc-AKR7A3-1	ncRNA_exonic	prognostic

Table S7. Functions of genes with prognostic DESs shared by male and female GBMs.

Gene	Description	REF:
MMACHC	MMACHC is responsible for the binding and intracellular trafficking of cobalamin (vitamin B12). Elevated levels of plasma cobalamin were associated with increased cancer risk and poor prognosis.	PMID: 26724465<br>PMID: 30642843
H2AFV	H2AFV is an oncogenic histone variant that is overexpressed in cancers. It plays vital roles in the occurrence and progression of liver cancer	PMID: 32547065
STEEP1	STING is essential for control of infections and for tumor immunosurveillance, but it can also drive pathological inflammation. STEEP mediates STING ER exit and activation of signaling	PMID: 32690950
MRI1	Elevated expression of the encoded protein is associated with metastatic melanoma and this protein promotes melanoma cell invasion independent of its enzymatic activity.	https://www.ncbi.nlm.nih.gov/gene/84245
PGPEP1	The activity of PGPEP1 negatively correlated with progression of colorectal cancer	PMID: 26078706

Table S8. GO and KEGG analysis of 117 prognostic genes that were connected in the PPI network.

#term ID	term description	false discovery rate
GO:0034641	cellular nitrogen compound metabolic process	5.30E-08
GO:0008152	metabolic process	9.25E-08
GO:0044237	cellular metabolic process	9.65E-08
GO:0051649	establishment of localization in cell	1.65E-07
GO:0046483	heterocycle metabolic process	1.93E-07
GO:0046907	intracellular transport	1.93E-07
GO:0072594	establishment of protein localization to organelle	4.47E-07
GO:0006725	cellular aromatic compound metabolic process	7.34E-07
GO:1901360	organic cyclic compound metabolic process	7.34E-07
GO:0006605	protein targeting	1.14E-06
GO:0006612	protein targeting to membrane	1.14E-06
GO:0006614	SRP-dependent cotranslational protein targeting to membrane	1.14E-06
GO:0051641	cellular localization	1.16E-06
GO:0006139	nucleobase-containing compound metabolic process	1.46E-06
GO:0006886	intracellular protein transport	1.46E-06
GO:0000956	nuclear-transcribed mRNA catabolic process	3.38E-06
GO:0090150	establishment of protein localization to membrane	1.02E-05
GO:0006807	nitrogen compound metabolic process	1.23E-05
GO:0044271	cellular nitrogen compound biosynthetic process	1.56E-05
GO:0071704	organic substance metabolic process	2.16E-05
GO:0045184	establishment of protein localization	2.48E-05
GO:0043603	cellular amide metabolic process	2.76E-05
GO:0015031	protein transport	2.95E-05
GO:0019083	viral transcription	2.95E-05
GO:0034645	cellular macromolecule biosynthetic process	2.95E-05
GO:0044270	cellular nitrogen compound catabolic process	2.95E-05
GO:0046700	heterocycle catabolic process	2.95E-05
GO:0034655	nucleobase-containing compound catabolic process	3.86E-05
GO:0010467	gene expression	4.85E-05
GO:0090304	nucleic acid metabolic process	5.33E-05
GO:0044238	primary metabolic process	5.50E-05
GO:1901361	organic cyclic compound catabolic process	8.38E-05
GO:0034613	cellular protein localization	0.0001
GO:0006413	translational initiation	0.00011
GO:0006412	translation	0.00016
GO:0006518	peptide metabolic process	0.00016

GO:0043604	amide biosynthetic process	0.0002
GO:0008104	protein localization	0.00027
GO:0000184	nuclear-transcribed mRNA catabolic process, nonsense-mediated decay	0.0003
GO:0044249	cellular biosynthetic process	0.0003
GO:0071705	nitrogen compound transport	0.0003
GO:0016032	viral process	0.00032
GO:0044403	symbiotic process	0.00034
GO:0072657	protein localization to membrane	0.00061
GO:0016070	RNA metabolic process	0.00064
GO:0033036	macromolecule localization	0.00064
GO:0044265	cellular macromolecule catabolic process	0.00074
GO:0090305	nucleic acid phosphodiester bond hydrolysis	0.00075
GO:1901576	organic substance biosynthetic process	0.00075
GO:0071702	organic substance transport	0.00092
GO:0044248	cellular catabolic process	0.0012
GO:0043170	macromolecule metabolic process	0.0017
GO:0044260	cellular macromolecule metabolic process	0.0017
GO:0009056	catabolic process	0.0021
GO:0051234	establishment of localization	0.0025
GO:0010608	posttranscriptional regulation of gene expression	0.0026
GO:0006810	transport	0.0027
GO:0022613	ribonucleoprotein complex biogenesis	0.0027
GO:1901575	organic substance catabolic process	0.003
GO:0016071	mRNA metabolic process	0.0036
GO:0034470	ncRNA processing	0.0051
GO:0009987	cellular process	0.0071
GO:0006364	rRNA processing	0.0114
GO:0042254	ribosome biogenesis	0.0171
GO:0000462	maturation of SSU-rRNA from tricistronic rRNA transcript (SSU-rRNA, 5.8S rRNA, LSU-rRNA)	0.0177
GO:0051179	localization	0.0197
GO:1903624	regulation of DNA catabolic process	0.0273
GO:0061418	regulation of transcription from RNA polymerase II promoter in response to hypoxia	0.0278
GO:1901796	regulation of signal transduction by p53 class mediator	0.0278
GO:1901564	organonitrogen compound metabolic process	0.03
GO:1901566	organonitrogen compound biosynthetic process	0.03
GO:0006396	RNA processing	0.0385

GO:0006913	nucleocytoplasmic transport	0.0419
GO:0031329	regulation of cellular catabolic process	0.0481
hsa04217 (KEGG)	Necroptosis	0.0093
PMID:29129909	(2017) RNA editing by ADAR1 leads to context-dependent transcriptome-wide changes in RNA secondary structure.	0.005

Table S9. Editing sites and related information of genes editing-regulated, prognostic genes (Figure 6c, d) in TCGA.

ENSEMBL	refGene	Editing site	HR	p.adj	median editing difference (high risk -low risk)	p.adj	Comparison
**ENSG00000182180**	MRPS16	chr10_73250084-	5.82	0.043	0.10	0.0004	F1
**ENSG00000151176**	PLBD2	chr12_113390121+	4.39	0.096	0.08	0.0007	F1
**ENSG00000075415**	SLC25A3	chr12_98605171+	7.79	0.039	0.13	0.0008	F1
**ENSG00000119711**	ALDH6A1	chr14_74060242-	3.88	0.088	0.15	0.0009	F1
**ENSG00000102910**	LONP2	chr16_48354334+	4.24	0.068	0.09	0.0237	F1
**ENSG00000161533**	ACOX1	chr17_75943979-	3.67	0.108	0.09	0.0003	F1
**ENSG00000105197**	TIMM50	chr19_39491748+	3.81	0.100	0.10	0.0052	F1
**ENSG00000134086**	VHL	chr3_10153100+	3.27	0.142	0.05	0.0157	F1
**ENSG00000144791**	LIMD1	chr3_45680999+	3.58	0.116	0.12	0.0004	F1
**ENSG00000145029**	NICN1	chr3_49424123-	3.18	0.133	0.15	0.0410	F1
**ENSG00000163626**	COX18	chr4_73057182-	3.21	0.121	0.11	0.0041	F1
**ENSG00000149100**	EIF3M	chr11_32604346+	7.00	0.039	0.14	0.0012	F1
**ENSG00000111358**	GTF2H3	chr12_123661870+	4.17	0.069	0.03	0.0020	F1
**ENSG00000247077**	PGAM5	chr12_132721879+	3.16	0.133	0.03	0.0278	F1
**ENSG00000119616**	FCF1	chr14_74736291+	3.86	0.096	0.16	0.0014	F1
**ENSG00000144231**	POLR2D	chr2_127843852-	3.51	0.119	0.05	0.0011	F1
**ENSG00000197756**	RPL37A	chr2_216501921+	3.10	0.148	0.03	0.0133	F1
**ENSG00000055332**	EIF2AK2	chr2_37100534-	3.44	0.100	0.05	0.0094	F1
**ENSG00000088888**	MAVS	chr20_3871007+	4.90	0.047	0.07	0.0226	F1
**ENSG00000142166**	IFNAR1	chr21_33357727+	3.05	0.142	0.10	0.0064	F1
**ENSG00000100028**	SNRPD3	chr22_24576495+	3.07	0.148	0.11	0.0400	F1
**ENSG00000120253**	NUP43	chr6_149725409-	5.46	0.047	0.11	0.0399	F1
**ENSG00000105968**	H2AFV	chr7_44833371-	5.07	0.050	0.08	0.0002	F1
**ENSG00000101966**	XIAP	chrX_123912479+	3.56	0.118	0.08	0.0004	F1
**ENSG00000102158**	MAGT1	chrX_77828400-	3.16	0.133	0.05	0.0010	F1
**ENSG00000182180**	MRPS16	chr10_73249083-	0.24	0.079	-0.05	0.0000	M1
**ENSG00000151366**	NDUFC2	chr11_78068542-	0.28	0.080	-0.07	0.0001	M1
**ENSG00000075415**	SLC25A3	chr12_98603976+	0.34	0.078	-0.23	0.0000	M1
**ENSG00000161533**	ACOX1	chr17_75944122-	0.24	0.078	-0.14	0.0000	M1
**ENSG00000105197**	TIMM50	chr19_39491660+	0.32	0.077	-0.07	0.0000	M1
**ENSG00000134086**	VHL	chr3_10152659+	0.37	0.080	-0.05	0.0004	M1
**ENSG00000114054**	PCCB	chr3_136331408+	0.28	0.078	-0.12	0.0005	M1

**ENSG00000151923**	TIAL1	chr10_119573235-	2.48	0.148	-0.09	0.0406	M1
**ENSG00000149100**	EIF3M	chr11_32604186+	0.26	0.082	-0.14	0.0000	M1
**ENSG00000119616**	FCF1	chr14_74735609+	0.33	0.143	-0.07	0.0000	M1
**ENSG00000144231**	POLR2D	chr2_127846010-	0.40	0.149	-0.11	0.0292	M1
**ENSG00000197756**	RPL37A	chr2_216502063+	0.36	0.078	-0.08	0.0011	M1
**ENSG00000055332**	EIF2AK2	chr2_37103895-	0.27	0.110	-0.18	0.0000	M1
**ENSG00000120253**	NUP43	chr6_149725204-	0.35	0.080	-0.06	0.0042	M1
**ENSG00000105968**	H2AFV	chr7_44833263-	0.23	0.077	-0.07	0.0000	M1
**ENSG00000101966**	XIAP	chrX_123913320+	0.24	0.078	-0.10	0.0000	M1
**ENSG00000114054**	PCCB	chr3_136331845+	0.23	0.146	-0.12	0.0000	M2
**ENSG00000247077**	PGAM5	chr12_132721754+	0.24	0.126	-0.03	0.0008	M2
**ENSG00000101966**	XIAP	chrX_123911555+	0.27	0.126	-0.04	0.0000	M2
**ENSG00000119711**	ALDH6A1	chr14_74058071-	12.84	0.133	0.17	0.0004	F3
**ENSG00000184575**	XPOT	chr12_64448979+	41.91	0.057	0.08	0.0181	F3
**ENSG00000129566**	TEP1	chr14_20366228-	6.73	0.120	0.11	0.0121	F3
**ENSG00000100028**	SNRPD3	chr22_24574605+	40.90	0.120	0.06	0.0003	F3

Table S10. HRs of editing-regulated, prognostic genes (Fig.6) in CGGA.

	ENSEMBL	refGene	age-adj HR	p.value
Males	ENSG00000120253	NUP43	0.174	0.00007
	ENSG00000129566	TEP1	0.169	0.00024
	ENSG00000151366	NDUFC2	0.260	0.00034
	ENSG00000119711	ALDH6A1	0.251	0.00183
	ENSG00000151923	TIAL1	4.039	0.00252
	ENSG00000055332	EIF2AK2	0.274	0.00300
	ENSG00000197756	RPL37A	0.289	0.00364
	ENSG00000101966	XIAP	0.393	0.00367
	ENSG00000144791	LIMD1	0.294	0.00368
	ENSG00000105968	H2AFV	0.322	0.00391
	ENSG00000161533	ACOX1	0.375	0.00425
	ENSG00000088888	MAVS	0.300	0.00550
	ENSG00000102910	LONP2	0.386	0.00580
	ENSG00000102158	MAGT1	0.414	0.00612
	ENSG00000134086	VHL	2.406	0.00919
	ENSG00000149100	EIF3M	2.649	0.01178
	ENSG00000114054	PCCB	0.267	0.01446
	ENSG00000100028	SNRPD3	0.380	0.01567
	ENSG00000142166	IFNAR1	0.477	0.02077
	ENSG00000075415	SLC25A3	0.377	0.02155
	ENSG00000105197	TIMM50	0.463	0.03108
	ENSG00000151176	PLBD2	0.447	0.03118
	ENSG00000184575	XPOT	0.506	0.03433
	ENSG00000247077	PGAM5	0.426	0.03751
	ENSG00000145029	NICN1	0.466	0.03882
	ENSG00000119616	FCF1	0.424	0.04269
Females	ENSG00000088888	MAVS	8.380	0.00094
	ENSG00000151176	PLBD2	17.122	0.00145
	ENSG00000100028	SNRPD3	6.861	0.00191
	ENSG00000075415	SLC25A3	11.792	0.00559
	ENSG00000120253	NUP43	8.596	0.00608
	ENSG00000161533	ACOX1	7.452	0.00760
	ENSG00000144791	LIMD1	6.872	0.00785
	ENSG00000111358	GTF2H3	4.185	0.01005
	ENSG00000105968	H2AFV	6.569	0.01170
	ENSG00000055332	EIF2AK2	5.779	0.01219

ENSG00000142166	IFNAR1	4.871	0.01458
ENSG00000149100	EIF3M	16.499	0.01552
ENSG00000182180	MRPS16	0.199	0.01766
ENSG00000247077	PGAM5	4.421	0.01963
ENSG00000151366	NDUFC2	3.697	0.02786
ENSG00000102158	MAGT1	4.607	0.03077
ENSG00000101966	XIAP	3.974	0.03287
ENSG00000119616	FCF1	3.735	0.03632
ENSG00000151923	TIAL1	10.182	0.03841
ENSG00000163626	COX18	3.182	0.03947
ENSG00000105197	TIMM50	3.581	0.04022
ENSG00000134086	VHL	3.572	0.04544

Table S11. Correlation of editing-regulated, prognostic genes (Fig.6) in CGGA.

	**ENSEMBL**	**refGene**	**age-adj R2**	***p*.value**
Males	ENSG00000134086	VHL	0.2950	0.0000
	ENSG00000151366	NDUFC2	0.2508	0.0002
	ENSG00000144791	LIMD1	0.2182	0.0004
	ENSG00000055332	EIF2AK2	0.2036	0.0007
	ENSG00000188846	RPL14	0.2899	0.0008
	ENSG00000129566	TEP1	0.1977	0.0018
	ENSG00000105968	H2AZ2	0.1805	0.0020
	ENSG00000182180	MRPS16	0.1437	0.0030
	ENSG00000151176	PLBD2	0.1392	0.0043
	ENSG00000101966	XIAP	0.2070	0.0051
	ENSG00000120253	NUP43	0.1326	0.0062
	ENSG00000161533	ACOX1	0.1211	0.0065
	ENSG00000119711	ALDH6A1	0.1269	0.0075
	ENSG00000088888	MAVS	0.1187	0.0093
	ENSG00000075415	SLC25A3	0.1610	0.0104
	ENSG00000102158	MAGT1	0.1089	0.0105
	ENSG00000145029	NICN1	0.1205	0.0115
	ENSG00000197756	RPL37A	0.0982	0.0154
	ENSG00000151923	TIAL1	0.1125	0.0186
	ENSG00000102910	LONP2	0.0766	0.0311
	ENSG00000119616	FCF1	0.0645	0.0314
	ENSG00000114054	PCCB	0.0654	0.0343
	ENSG00000100028	SNRPD3	0.0739	0.0408
	ENSG00000105197	TIMM50	0.0642	0.0423
Females	ENSG00000247077	PGAM5	0.3873	0.0007
	ENSG00000075415	SLC25A3	0.4200	0.0008
	ENSG00000142166	IFNAR1	0.4309	0.0010
	ENSG00000105968	H2AZ2	0.3323	0.0015
	ENSG00000101966	XIAP	0.3283	0.0016
	ENSG00000161533	ACOX1	0.3214	0.0022
	ENSG00000102910	LONP2	0.3830	0.0022
	ENSG00000088888	MAVS	0.3566	0.0043
	ENSG00000102158	MAGT1	0.2715	0.0046
	ENSG00000151176	PLBD2	0.3174	0.0050
	ENSG00000119711	ALDH6A1	0.3655	0.0059
	ENSG00000055332	EIF2AK2	0.3169	0.0060

ENSG00000163626	COX18	0.2397	0.0062
ENSG00000134086	VHL	0.1924	0.0142
ENSG00000144791	LIMD1	0.1685	0.0232
ENSG00000105197	TIMM50	0.2153	0.0260
ENSG00000182180	MRPS16	0.1691	0.0319
ENSG00000149100	EIF3M	0.2213	0.0342
ENSG00000197756	RPL37A	0.1166	0.0358
ENSG00000120253	NUP43	0.2726	0.0514

Reference

1. Silvestris, D.A.; Picardi, E.; Cesarini, V.; Fosso, B.; Mangraviti, N.; Massimi, L.; Martini, M.; Pesole, G.; Locatelli, F.; Gallo, A. Dynamic inosinome profiles reveal novel patient stratification and gender-specific differences in glioblastoma. *Genome Biol.* **2019**, *20*, 33.
